# Genome Identification of *GLP* Family in Korean Pine and Study on the Function of *GLP1-2-6*/*GLP1-2-21* in Somatic Embryo Maturation

**DOI:** 10.3390/plants15030476

**Published:** 2026-02-03

**Authors:** Shuoran Tang, Ling Yang

**Affiliations:** 1 College of Forestry, Beijing Forestry University, Beijing 100091, China; tangshuoran2022@163.com; 2 State Key Laboratory of Tree Genetics and Breeding, Northeast Forestry University, Harbin 150040, China

**Keywords:** Korean pine, *GLP*, somatic embryo maturation, genetic transformation, abscisic acid

## Abstract

Based on prior transcriptome data, we established a core gene interaction network for Korean pine somatic embryo maturation and screened 18 core genes. These genes showed distinct differential expression in early somatic embryogenesis. In particular, *PkGLP1-2-6 (Pkor04G01180)* and *PkGLP-1-2-21 (Pkor04G01200)* were highly correlated in the network and can be regarded as key genes mediating Korean pine somatic embryo maturation. A total of 92 members of the *PkGLP* gene family were identified in the Korean pine genome, which can be classified into 8 subfamilies based on evolutionary relationships. Both *PkGLP1-2-6* and *PkGLP1-2-21* were localized in the cell membrane and nucleus. By means of a stable genetic transformation system, transgenic Korean pine calli overexpressing *PkGLP1-2-6* and *PkGLP1-2-21* were successfully established. The results demonstrated that the overexpression of *PkGLP1-2-6* and *PkGLP1-2-21* could effectively promote somatic embryogenesis and enhance the yield of somatic embryos. In the presence of exogenous abscisic acid (ABA), the somatic embryo yield of the transgenic lines was significantly higher than that of the wild-type controls. Compared with the wild-type controls, the SOD activity in the cell lines overexpressing *PkGLP1-2-6* and *PkGLP1-2-21* was significantly increased, whereas the activities of POD and CAT were decreased, and the contents of H_2_O_2_ and superoxide anion (O_2_^−^) were significantly reduced. These results indicate that *PkGLP1-2-6* and *PkGLP1-2-21* are actively involved in the reactive oxygen species (ROS) scavenging process during somatic embryogenesis of Korean pine. The overexpression of *PkGLP1-2-6* and *PkGLP1-2-21* contributes to enhancing the antioxidant capacity of cells, thereby increasing the yield of somatic embryos.

## 1. Introduction

Germin-like proteins (*GLP*s) are ubiquitous in various plant species, including monocots such as wheat (*Triticum aestivum*) [1], barley (*Hordeum vulgare*) [1], maize (*Zea mays*) [2], and rice (Oryza sativa) [3]. They are also present in dicotyledonous plants such as *Arabidopsis thaliana* [4], *Vitis vinifera* [5], and *Glycine max* [5], as well as in gymnosperms including the genus *Pinus* [6]. *GLP* exhibits distinct functions at different plant developmental stages [7] and is primarily involved in a wide range of physiological processes, acting as receptors, enzymes and structural proteins. Notable progress has been made in research on plant resistance to biotic stress [8]. Overexpression of *GLP* promotes plant growth and development by modulating ROS metabolism and stress-responsive pathways. In studies focusing on the molecular regulation of plant somatic embryogenesis, *GLP* has been found to localize in the extracellular matrix, exhibit peroxidase activity under specific conditions, and participate in cell wall metabolism. In the longan (*Dimocarpus longan*) cell line overexpressing *DlGLP1-5-1*, *DlGLP1-5-1* was found to regulate the H_2_O_2_ content in plants [9]. In *Arabidopsis thaliana*, transgenic lines overexpressing *AtGLP* exhibited longer roots and superior development compared to the wild type following salt stress [10], and their fresh weight was also significantly higher than that of the wild type [11]. Additionally, *GLP* is a key factor in plant somatic embryogenesis and can serve as a marker protein for early-stage somatic embryogenesis [12]. Using the previously obtained transcriptome data [13], we constructed a core gene interaction network associated with the maturation of Korean pine somatic embryos, from which a total of 18 core genes were identified (Figure S1). Among them, *Pkor04G01200* and *Pkor04G01180* with high connectivity encode *GLP*, which is a key factor in the process of plant somatic embryogenesis. *GLP* genes exhibited significant differential expression during the early stage of somatic embryogenesis in Korean pine. Based on these findings, we hypothesized that *PkGLP1-2-6* (*Pkor04G01180*) and *PkGLP-1-2-21* (*Pkor04G01200*) exert crucial regulatory effects on the maturation of somatic embryos.

To further explore the potential role of *GLP* in Korean pine, this study identified the *GLP* gene family across the Korean pine genome, analyzed its phylogenetic relationships, conserved motifs, gene structural characteristics, and expression patterns under abiotic stress, and verified its functional properties. Subcellular localization analysis of *PkGLP1-2-6* and *PkGLP1-2-21* proteins was performed, and their cellular distribution characteristics were preliminarily clarified, laying a foundation for subsequent functional studies. Overexpression vectors of *PkGLP1-2-6* and *PkGLP1-2-21* were constructed, and transgenic calli were obtained via the stable genetic transformation system of Korean pine, followed by molecular identification of the transgenic calli. Somatic embryos were cultured in the presence or absence of exogenous ABA, and the number of somatic embryos was quantified. The role of *PkGLP1-2-6* and *PkGLP1-2-21* in Korean pine somatic embryo maturation was further validated, thereby providing new insights into the function of the *GLP* family in Korean pine somatic embryogenesis.

## 2. Results

### 2.1. Construction of Weighted Gene Co-Expression Network

A weighted gene co-expression network was constructed using genes with low expression variation from the filtered expression matrix. When the soft threshold β = 28, the scale-free network fitting index R^2^ > 0.8 and the average connectivity tends to 0; thus, this parameter was selected for scale-free network construction (Figure S2). Subsequently, genes were subjected to hierarchical clustering and divided into modules, followed by the calculation of each module’s feature vector (module eigengene) and merging of highly similar modules. A total of six ABA response-specific gene co-expression modules were identified based on the criteria of |r| > 0.50 and *p* < 0.05. Among these modules, the blue module exhibited a strong positive correlation with somatic embryos cultured for 7 days under exogenous ABA treatment during maturation (Figure S3). The gene regulatory network of the blue module (corresponding to 7-day exogenous ABA-treated somatic embryo maturation culture) was visualized using Cytoscape software (v3.10.4). From this network, 18 genes with the highest connectivity were identified and designated as the core regulatory genes of the module, and the gene interaction network diagram was generated (Figure S1). Notably, two of these high-connectivity genes, *PkGLP1-2-6* (*Pkor04G01180*) and *PkGLP1-2-21* (*Pkor04G01200*), encode germin-like proteins (*GLP*s)—key factors involved in plant somatic embryogenesis.

### 2.2. Identification and Physicochemical Properties Analysis of PkGLP Gene Family Members

**Figure 1:**
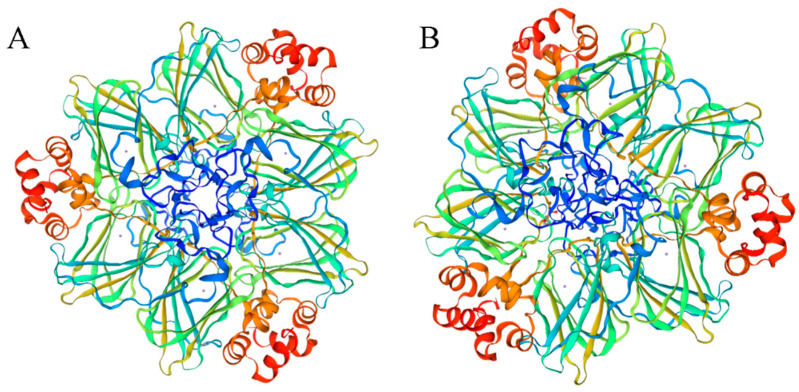
Tertiary structure of *PkGLP1-2-6* and *PkGLP1-2-21*. (**A**): *PkGLP1-2-6*; (**B**): *PkGLP1-2-21*.

A total of 92 *PkGLP* gene family members harboring the complete conserved Cupin-1 domain were identified from the genome of Korean pine via genome-wide screening. The protein lengths of *PkGLP* family members ranged from 125 to 244 amino acids (Table S1), with molecular weights of 13.39–26.05 kDa and isoelectric points (pI) of 4.87–9.59. Subcellular localization prediction analysis indicated that the majority of *PkGLP* family members were localized to the extracellular matrix. The α-helix content of the 92 *PkGLP* members ranged from 14.56% to 24.96%, and the majority of *PkGLP* proteins contained signal peptides. The tertiary structure prediction showed that both *PkGLP1-2-6* and *PkGLP1-2-21* had OXO activity (Figure 1).

### 2.3. Family Phylogenetic Tree of PkGLP Gene Family

**Figure 2:**
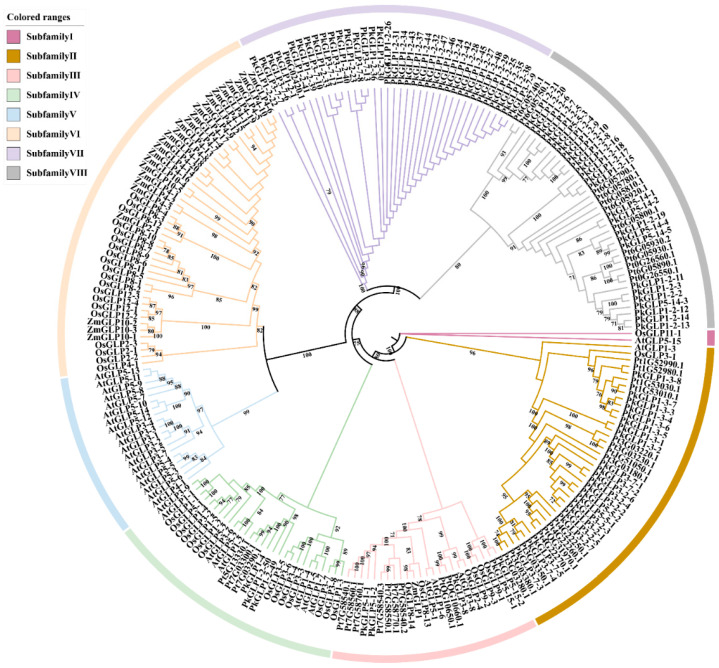
Phylogenetic tree of *GLP* family members in Korean pine (Pk), *Arabidopsis thaliana* (At), rice (Os), maize (Zm) and *Pinus tabulaeformis* (Pt).

To investigate the evolutionary relationships of *PkGLP* genes, a phylogenetic tree was constructed encompassing 234 *GLP* members, including 92 from Korean pine, 32 from *Arabidopsis thaliana*, 43 from rice (*Oryza sativa*), 27 from maize (*Zea mays*), and 40 from *Pinus tabulaeformis* (Figure 2). The 234 *GLP* genes were clustered into eight subfamilies, designated as Subfamilies I to VIII. *PkGLP* is mainly distributed in subfamilies II, III, VII and VIII.

### 2.4. Chromosome Location and Collinearity Analysis of PkGLP Gene Family

**Figure 3:**
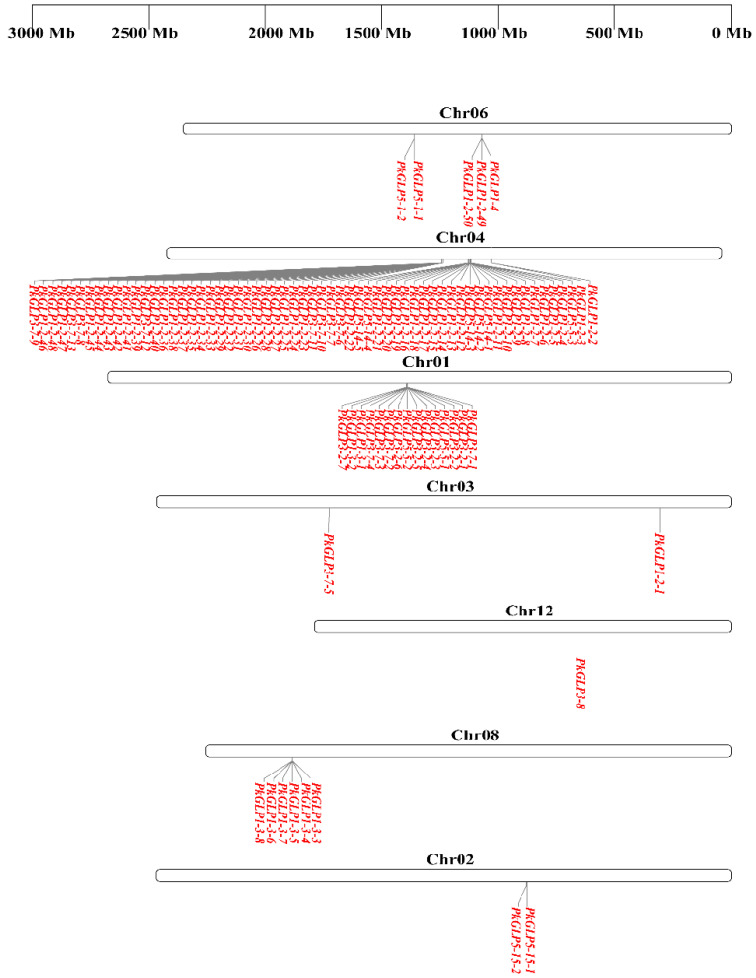
Chromosomal localization of *PkGLP* gene family.

Chromosomal localization analysis revealed that the 92 *PkGLP* genes were unevenly distributed across chromosomes 1–12 of Korean pine (Figure 3). Gene duplication represents one of the key mechanisms driving genome evolution [14], and tandem duplication is recognized as a major pathway underlying the expansion of plant gene families [15]. A typical characteristic of this process is that multiple members of the same gene family are clustered in the same or adjacent genomic regions [16]. Tandem duplication events of *PkGLP* genes were detected on chromosomes chr01, chr02, chr04, chr06, and chr08. Notably, 61 *PkGLP* genes were concentrated on chromosome Chr04, among which 25 and 35 genes formed two distinct gene clusters, respectively.

**Figure 4:**
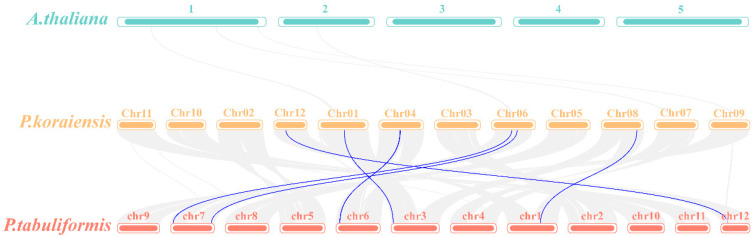
Colinearity analysis of *GLP* genes in Korean pine, *Arabidopsis thaliana* and *Pinus tabulaeformis*. 1–5, 5 chromosomes of *Arabidopsis thaliana*; chr01–chr12, 12 chromosomes of Korean pine; chr1–chr12, 12 chromosomes of *Pinus tabuliformis*.

To further investigate the evolutionary relationships within the *PkGLP* family, genome collinearity analysis was performed between *Arabidopsis thaliana*, Korean pine, and *Pinus tabuliformis* using McScan X under default parameters (Figure 4). The results indicated that no collinear relationships existed between the *GLP* genes of Korean pine and *Arabidopsis thaliana*. In contrast, 6 *GLP* gene pairs were identified in the collinear genomic regions between Korean pine and *Pinus tabuliformis*, demonstrating significant collinear relationships between these two coniferous species. The results demonstrated that the identified *PkGLP* genes from Korean pine were directly homologous to the *PtGLP* genes from *Pinus tabuliformis*.

### 2.5. Conserved Motif and Gene Structure Analysis of PkGLP Gene Family

Conserved motif and gene structure analyses revealed that each *PkGLP* family member contained 4 to 8 conserved motifs, with intron numbers ranging from 0 to 3 (Figure S4). The results showed that the tissue form of the conserved motifs of *GLP*s within the same subfamily showed high consistency, indicating that *GLP*s members within the same subfamily may undertake similar biological functions, which indirectly reflects the reliability of phylogenetic analysis. All *PkGLP* family members harbored Motif 3 and Motif 4. With the exceptions of *PkGLP5-15-1* and *PkGLP5-15-2* (which lacked Motif 2 and Motif 5) and *PkGLP5-5-3* (which lacked Motif 5), all the remaining members contained both Motif 2 and Motif 5. Integrated analysis revealed that all members within the same subfamily exhibited similar conserved motif compositions and gene structural features. In summary, the structural characteristics of each member within the same *PkGLP* subfamily are highly consistent, indicating that these members may share conserved biological functions.

### 2.6. Analysis of Cis-Acting Elements of PkGLP Promoter

**Figure 5:**
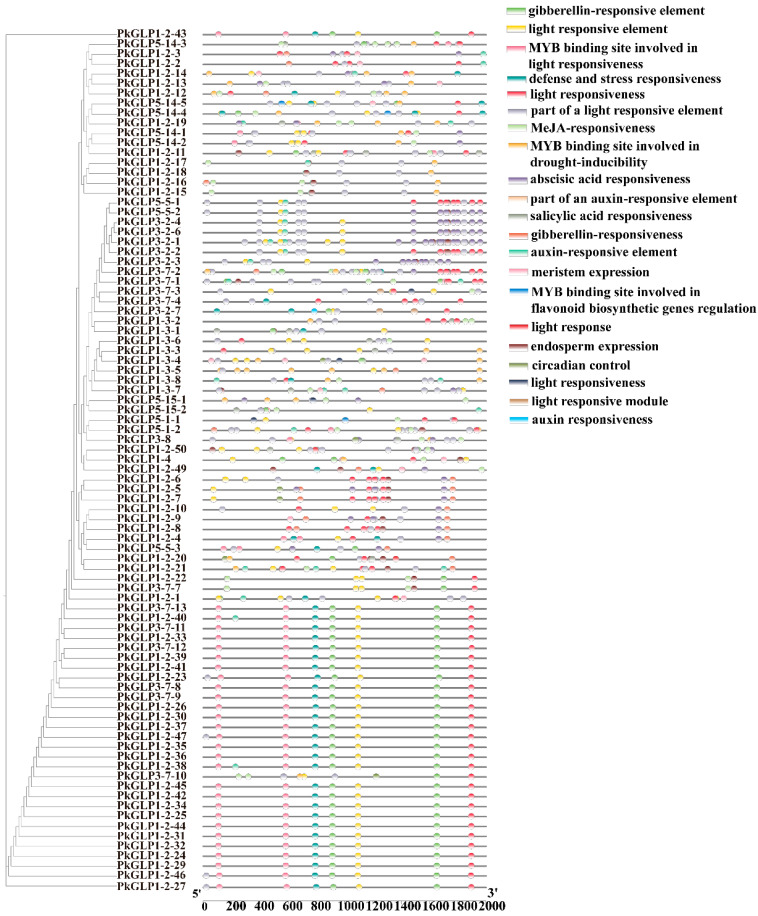
Distribution of cis-acting elements of *PkGLP* family members.

To further investigate the regulatory mechanisms of *PkGLP* genes in Korean pine, cis-acting elements within the 2000 bp upstream region of the translation initiation site were systematically predicted using the PlantCARE database (Figure 5). The results revealed that the promoters of *PkGLP* genes contained abundant hormone-responsive elements, including those responsive to auxin, abscisic acid (ABA), gibberellin (GA), methyl jasmonate (MeJA), and salicylic acid (SA). A substantial number of stress-responsive elements were also identified, with a particular enrichment of those associated with drought tolerance and defense responses. Additionally, tissue-specific regulatory elements associated with meristem development and endosperm-specific expression were also detected. Collectively, these findings indicate that *PkGLP* genes may serve as key regulators involved in hormone signaling pathways, environmental stress responses, and growth and development processes of Korean pine, thereby providing new insights into their regulatory roles in conifer biology.

### 2.7. Expression Analysis of PkGLP Gene Under Abiotic Stress

**Figure 6:**
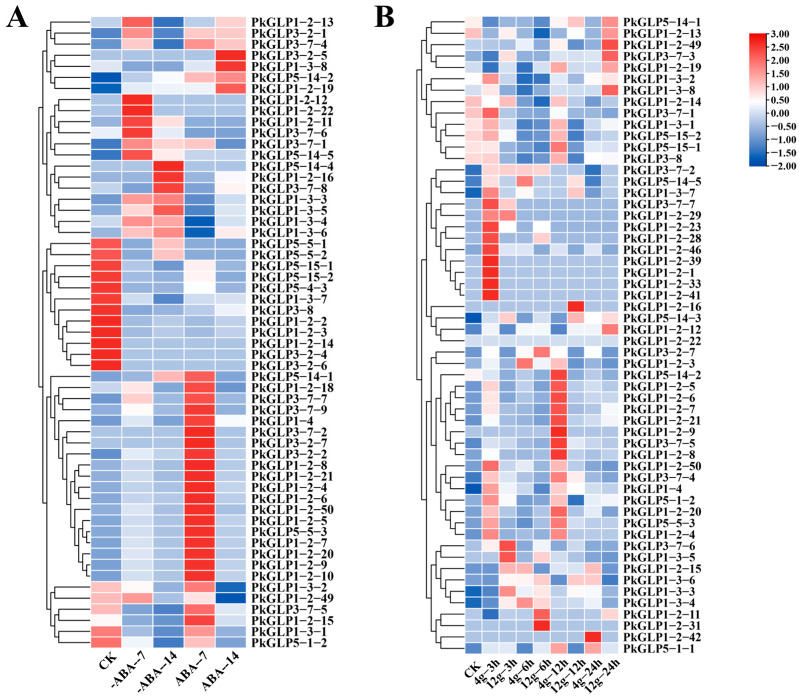
Expression analysis of *PkGLP* gene under abiotic stress. (**A**) Expression analysis of *PkGLP* gene during the maturation of lower body embryos with or without exogenous ABA of 80 μmol/L; (**B**) Expression analysis of *PkGLP* gene during the maturation of somatic embryo maturation under different concentrations of gellan gum.

Under treatments with different concentrations of ABA, *PkGLP* family members exhibited distinct differential expression patterns (Table S2, Figure 6A). The expression of certain *PkGLP* genes was upregulated under the induction of exogenous ABA, suggesting that these genes may affect the formation of somatic embryos in Korean pine. The expression levels of certain *PkGLP* genes were relatively high at 0 d (CK) of somatic embryo maturation culture, and subsequently decreased with the extension of culture duration. The expression levels of *PkGLP1-2-6* and *PkGLP1-2-21* were significantly increased on the 7th day of somatic embryo maturation under the action of exogenous ABA compared with those without addition. Under treatments with different concentrations of gellan gum, the expression patterns of *PkGLP* family members were visualized (Table S3, Figure 6B). Compared with the somatic embryo maturation process under the treatment of 4 g/L gellan gum, certain *PkGLP* genes exhibited high expression levels at 3 h and 12 h of culture. This expression pattern indicates that the transcription of some *PkGLP* genes may be downregulated under water stress conditions induced by gellan gum.

### 2.8. The qRT-PCR Verification of PkGLP Gene Under ABA Treatment

**Figure 7:**
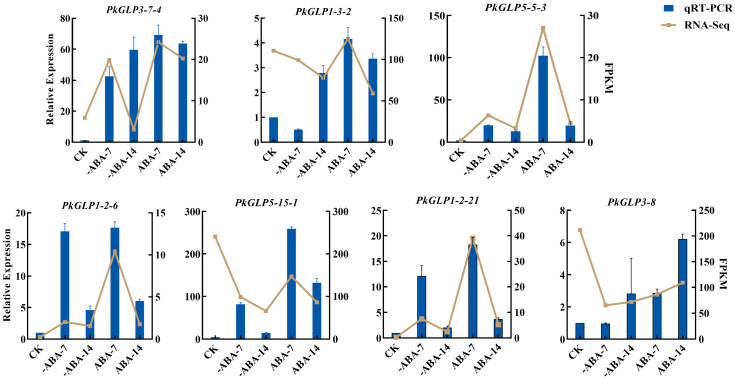
Expression analysis of *PkGLP* family members in the early stage of Korean pine somatic embryogenesis.

The effect of exogenous ABA treatment on the expression of *PkGLP* genes during the early somatic embryogenesis stage of Korean pine was verified via qRT-PCR. Based on transcriptome data analysis, seven *PkGLP* genes with significant differential expression during the early stage of somatic embryogenesis were screened out for subsequent verification. The results revealed that the expression levels of *PkGLP5-15-1*, *PkGLP5-5-3*, *PkGLP1-2-6* and *PkGLP1-2-21* genes were significantly upregulated on the 7th day of somatic embryo maturation culture, regardless of the presence or absence of exogenous ABA, whereas their expression levels decreased on the 14th day of culture. With the extension of somatic embryo maturation duration, the expression level of *PkGLP3-8* was also upregulated, which was consistent with its expression trend in the transcriptome data (Figure 7). Under the treatment of exogenous ABA, the expression levels of these seven *PkGLP* genes were significantly upregulated, indicating that these *PkGLP* genes may play important roles in the ABA response during somatic embryo maturation of Korean pine.

### 2.9. Subcellular Localization of PkGLP1-2-6 and PkGLP1-2-21 Proteins in Tobacco

**Figure 8:**
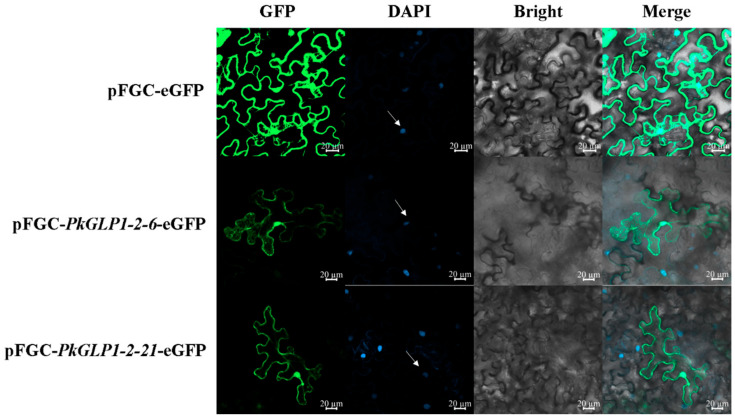
Subcellular localization of pFGC-*PkGLP1-2-6*-eGFP and pFGC-*PkGLP1-2-21*-eGFP. DAPI, a dye used to stain the nucleus; BF, bright field; Merge, merged images of BF, GFP, and DAPI staining. Scale bar = 20 µm.

The subcellular localization vectors pFGC-*PkGLP1-2-6*-eGFP and pFGC-*PkGLP1-2-21*-eGFP were successfully constructed by fusing the target *PkGLP* genes with the pFGC vector harboring the enhanced green fluorescent protein (eGFP) tag. Finally, Agrobacterium solutions containing the pFGC-*PkGLP1-2-6*-eGFP and pFGC-*PkGLP1-2-21*-eGFP vectors were successfully obtained. Subsequently, the subcellular localization of the fusion proteins was observed and analyzed using a laser scanning confocal microscope (LSCM). The results revealed that the empty vector pFGC-eGFP produced fluorescence signals in the cell membrane, cytoplasm, and nucleus of tobacco leaf cells; in contrast, the recombinant vectors pFGC-*PkGLP1-2-6*-eGFP and pFGC-*PkGLP1-2-21*-eGFP exhibited GFP fluorescence signals exclusively in the nucleus and cell membrane (Figure 8). These findings indicate that the *PkGLP1-2-6* and *PkGLP1-2-21* proteins are localized in the nucleus and cell membrane.

### 2.10. Screening of PkGLP1-2-6 and PkGLP1-2-21 Transgenic Callus and Somatic Embryo Maturation Culture

**Figure 9:**
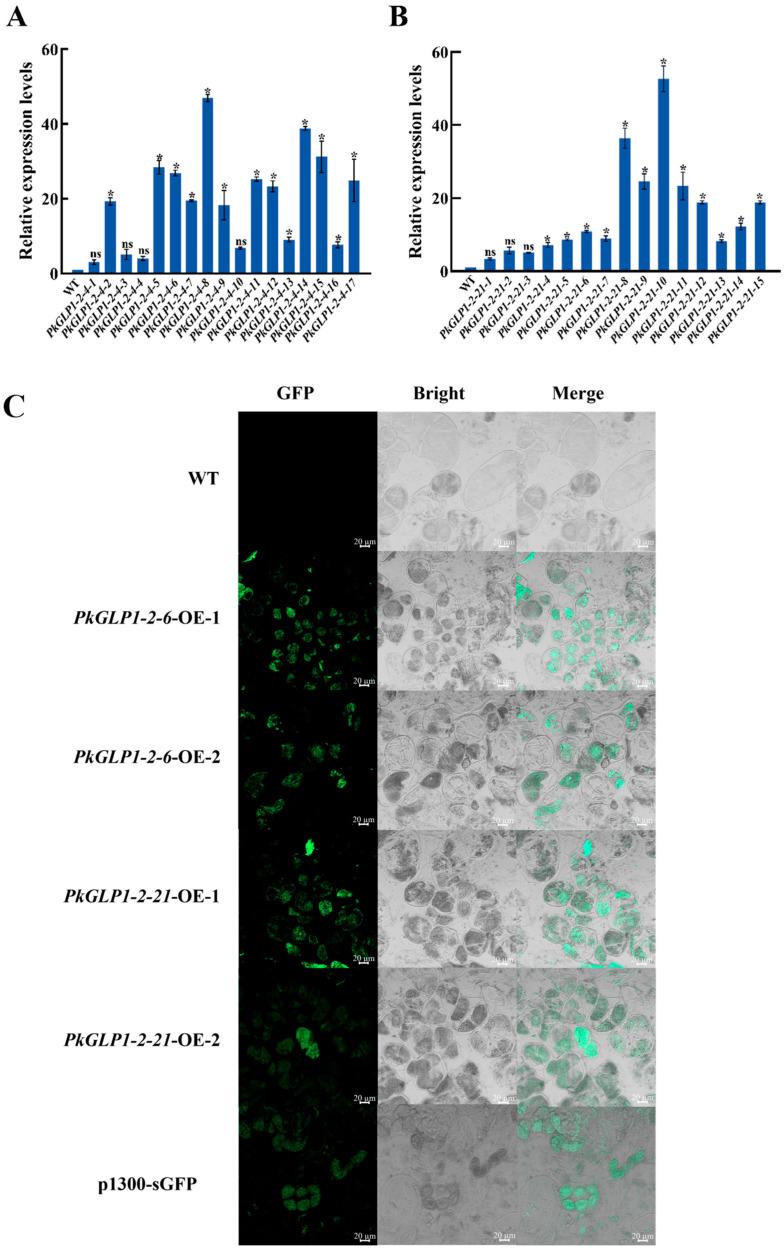
Identification of transgenic callus. (**A**): *PkGLP1-2-6* transgenic callus identification; (**B**): *PkGLP1-2-21* transgenic callus identification; (**C**): GFP fluorescence localization of wild type and pCambia1300-*35S*-eGFP transformation materials. The relative expression level was the mean and standard deviation of each group of treatments; *: *p* < 0.05 compared to wild type; ns: *p* > 0.05 compared to wild type.

To investigate the functions of *PkGLP1-2-6* and *PkGLP1-2-21* during somatic embryogenesis, the corresponding plant overexpression vectors were constructed in this study. Furthermore, several transgenic callus lines of Korean pine were obtained via stable genetic transformation. The expression levels of the target genes in the two transgenic callus lines were quantitatively analyzed via qRT-PCR. Four lines with high target gene expression levels were subsequently selected for follow-up experiments, namely *PkGLP1-2-6*-OE-1, *PkGLP1-2-6*-OE-2, *PkGLP1-2-21*-OE-1, and *PkGLP1-2-21*-OE-2 (Figure 9A,B). Wild-type explants and transgenic calli of *PkGLP1-2-6*-OE, *PkGLP1-2-21*-OE and p1300-sGFP were subjected to GFP fluorescence observation using a LSCM (Figure 9C). No GFP fluorescence signal was detected in the wild-type explants, whereas distinct green fluorescence signals were observed in the transgenic calli.

**Figure 10:**
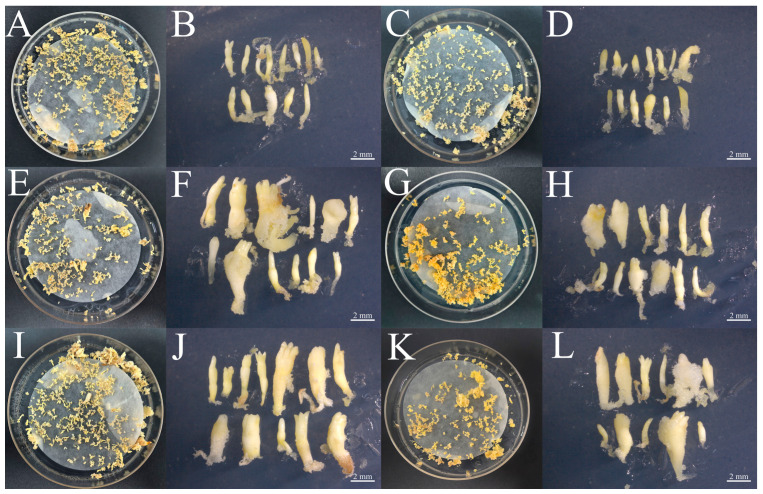
Wild type somatic embryos and *PkGLP1-2-6* and *PkGLP1-2-21* transgenic somatic embryos of Korean pine. (**A**): WT-a-1 mature material (80 μmol/LABA); (**B**): WT-a-1 somatic embryo (80 μmol/LABA); (**C**): WT-a-1 somatic embryo (0 μmol/L ABA); (**D**): WT-a-1 somatic embryo (0 μmol/LABA); (**E**): *PkGLP1-2-6*-OE mature material (80 μmol/L ABA); (**F**): *PkGLP1-2-6*-OE somatic embryo (80 μmol/L ABA); (**G**): *PkGLP1-2-6*-OE mature material (0 μmol/L ABA); (**H**): *PkGLP1-2-6*-OE somatic embryo (0 μmol/L ABA); (**I**): *PkGLP1-2-21*-OE mature material (80 μmol/L ABA); (**J**): *PkGLP1-2-21*-OE somatic embryo (80 μmol/L ABA); (**K**): *PkGLP1-2-21*-OE mature material (0 μmol/L ABA); (**L**): *PkGLP1-2-21*-OE somatic embryo (0 μmol/L ABA).

**Figure 11:**
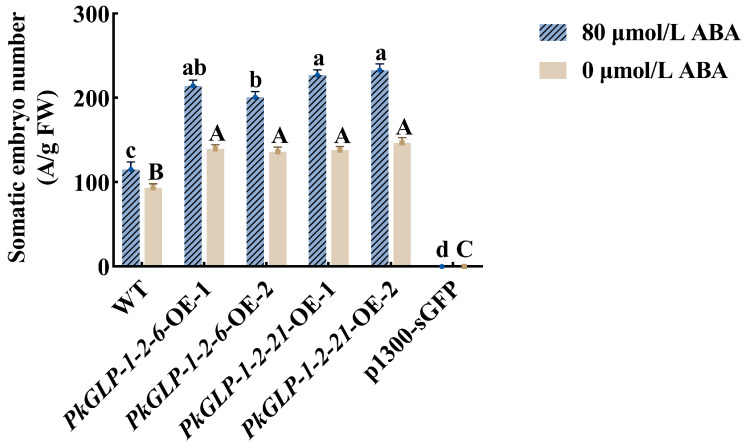
The number of wild-type and transgenic somatic embryos of Korean pine. Different lowercase letters indicate that different cell lines have significant differences at the level of *p* < 0.05 when 80 μmol/L ABA is added. Different capital letters indicate that there are significant differences in different sampling times at the level of *p* < 0.05 without ABA.

The *PkGLP1-2-6*-OE and *PkGLP1-2-21*-OE transgenic calli, together with the wild-type explant (a-1), were cultured synchronously for somatic embryo maturation. Subsequently, the number of somatic embryos was statistically counted (Figure 10 and Figure 11). Regardless of the presence or absence of exogenous ABA, the number of somatic embryos in the wild-type explants was lower than that in the *PkGLP1-2-6*-OE and *PkGLP1-2-21*-OE transgenic calli. No somatic embryos were produced in the empty strain with or without exogenous ABA. The results demonstrated that the overexpression of *PkGLP1-2-6* and *PkGLP1-2-21* genes significantly promotes the formation of somatic embryos in Korean pine.

### 2.11. Determination of Redox Substances in Wild-Type and Transgenic Callus and Somatic Embryos

**Figure 12:**
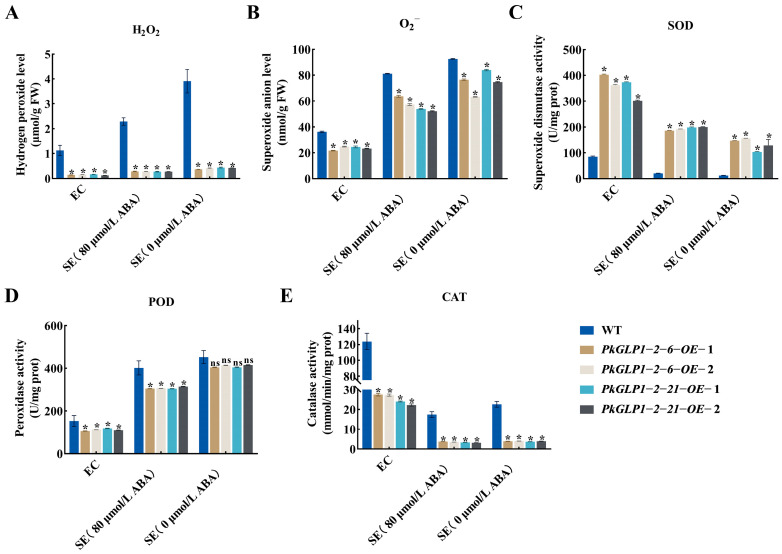
Changes in redox substances in wild-type and transgenic strains EC and SE of Korean pine. (**A**) H_2_O_2_ content; (**B**) O_2_^−^ content; (**C**) superoxide dismutase activity; (**D**) peroxidase activity; (**E**) catalase activity. * indicates that there is a significant difference between transgenic lines and wild type at the level of *p* < 0.05; ns indicates that the transgenic strain is *p* > 0.05 compared with the wild type.

The redox-related substance contents in the calli and somatic embryos of the wild-type line (a-1) and four transgenic lines were determined (Figure 12). Compared with the wild type (WT), the SOD activity in the *PkGLP*-overexpressing lines was significantly increased. The activities of POD and CAT were decreased, while the contents of H_2_O_2_ and O_2_^−^ were also significantly reduced. The results demonstrated that *PkGLP1-2-6* and *PkGLP1-2-21* play crucial roles in ROS scavenging during somatic embryogenesis of Korean pine, thereby facilitating somatic embryo formation and development.

## 3. Discussion

### 3.1. The Role of PkGLP Gene Family in Plant Growth and Development and Stress Response

*GLPs* are widely involved in multiple aspects of plant growth and development, as well as in the response to both biotic and abiotic stresses. To date, 32, 43, 27, and 40 *GLP* family members have been identified in *Arabidopsis thaliana*, *Oryza sativa*, maize (*Zea mays*), and *Pinus tabulaeformis*, respectively [17]. In this study, a genome-wide identification analysis was performed on the *GLP* family in Korean pine, and a total of 92 *PkGLP* family members were identified. By aligning the amino acid sequences of the 92 identified *PkGLP* family members, it was found that most of these proteins consist of 220 amino acids, with the exception of a small number of family members (e.g., *PkGLP1-2-1*) that exhibit sequence length variation. Most *PkGLP* genes encode proteins with typical signal peptide sequences, indicating that these proteins may be implicated in the biosynthesis and trafficking of intracellular and extracellular glycans and lipids [18]. The expansion of the *PkGLP* gene family is likely dominated by tandem duplication events, whereas variations in gene structure further contribute to the divergence of expression patterns and functional differentiation among its members [19,20]. Based on phylogenetic analysis, the *GLP* family members from Korean pine, *Arabidopsis thaliana*, *Oryza sativa*, *Zea mays*, and *Pinus tabulaeformis* were clustered into 8 distinct subfamilies. *GLP* genes may have evolved species-specific functions across different plant taxa, and certain members are even uniquely restricted to monocotyledonous plants. Analysis of the *PkGLP* gene structure revealed that the formation of *PkGLP* gene clusters localized on chromosome 4 may be attributed to recent tandem duplication events. Additionally, the coding region sequences of these duplicated genes share high sequence similarity, which suggests that their biological functions are evolutionarily conserved. Li [17] reported that *GLP* genes from rice (*Oryza sativa*) and *Arabidopsis thaliana* exhibit structural conservation within the same subfamily. *Oryza sativa OsGLP8-7* promotes lignin deposition by modulating H_2_O_2_ accumulation, thereby mitigating copper stress [21]; meanwhile, the overexpression of *OsGLP* genes also enhances heavy metal tolerance in *Arabidopsis thaliana* [22]. The overexpression of *Oryza sativa OsRGLP1* in tobacco significantly increases the H_2_O_2_ in leaves, which in turn enhances intercellular cross-linking to improve disease resistance [23]. The accumulation of H_2_O_2_ enhances intercellular cross-linking, thereby improving the disease resistance of *Oryza sativa* [24]. Collectively, these results indicate that *GLP* genes are multifunctional regulators involved in plant growth, development, and stress responses. However, research regarding the roles of *GLP* genes in somatic embryo development remains scarce, and thus further in-depth investigations are warranted.

### 3.2. The Role of PkGLP in the Early Stage of Somatic Embryogenesis of Korean Pine Embryogenic Callus and ABA Response

*GLP* is a plant-specific glycosylated protein that is widely involved in plant development, stress responses, and cellular signal transduction. During the early stage of somatic embryogenesis, *GLP*s exert crucial regulatory roles by modulating redox homeostasis, cell wall remodeling, and hormone signaling pathways. In a study focusing on *GLP*s from larch (*Larix gmelinii*), silencing of the *LmGER1* gene was found to suppress callus maturation and inhibit the formation of globular embryos [6]. During somatic embryogenesis of longan, several *DlGLP* genes exhibited high expression levels at the incomplete compacted embryogenic callus (*ICpEC*) stage and the early globular embryo (GE) stage, indicating that these *DlGLP* genes may play critical roles during the early phase of longan somatic embryogenesis. As enzymes possessing both SOD and OXO activities, *GLP* are capable of generating H_2_O_2_, which in turn participates in the regulation of cell wall structural proteins via the Ca^2+^ signaling pathway [25]. The functional characteristics of *GLP* genes have been widely reported in angiosperms, but there is still a lack of systematic comparison between angiosperm and gymnosperm, which hinders the understanding of the evolutionary conservation and species specificity of *GLP* regulation in plant embryonic development. However, based on the above results of some species, it can be concluded that the mode of action of *GLP*s in different species has certain commonalities and unique characteristics. *GLP* possess intrinsic SOD activity, which enables them to scavenge ROS and maintain the redox homeostasis essential for somatic embryogenesis. ROS act as both signaling molecules and toxic substances. *GLP* influence somatic embryo formation by modulating ROS levels: they inhibit the excessive accumulation of ROS to prevent programmed cell death (PCD) and promote the formation of embryonic cell clusters. However, in rice callus, neither the overexpression nor the knockout of *OsGLP1* had an impact on plantlet formation, whereas allergic necrosis was observed in the mature leaves of T0 generation plants after 6 months of culture [26,27]. This result reveals that *GLP* genes may exhibit functional diversity during somatic embryonic development across different plant species, whereas their specific regulatory mechanisms remain to be further elucidated.

Analysis of promoter cis-acting elements revealed that the promoter regions of *GLP* family members contain response elements for ABA and other phytohormones, indicating that *PkGLP* genes may be involved in phytohormone signaling pathways. Tandemly repeated genes exhibit differences in the cis-acting elements within their promoter regions, which may contribute to distinct expression patterns during different developmental stages of Korean pine somatic embryos. Previous studies have demonstrated that the expression levels of *PkGLP* family members vary in response to different concentrations of ABA. Certain *PkGLP* genes exhibit high expression levels upon induction by exogenous ABA, whereas others are highly expressed in the absence of exogenous ABA. This indicates that different *PkGLP* members have distinct ABA response mechanisms and exert divergent effects on somatic embryogenesis. *PkGLP1-2-6* and *PkGLP1-2-21* exhibited significantly elevated expression levels at 7 days post exogenous ABA treatment, suggesting that the upregulation of these genes under ABA regulation facilitates somatic embryo formation.

### 3.3. The Role of PkGLP1-2-6 and PkGLP1-2-21 in Growth and Metabolism

Subcellular localization analysis is conducive to clarifying the subcellular distribution of target proteins and provides a critical theoretical basis for elucidating their biological functions. In this study, a genome-wide identification of the *GLP* gene family in Korean pine revealed that most members are localized to the extracellular matrix, and these genes may be involved in the regulation of cell growth and metabolism [17]. Subcellular localization assays were further performed for the *PkGLP1-2-6* and *PkGLP1-2-21* proteins, which were found to be localized in both the cell membrane and nucleus. This dual localization likely harbors important biological significance, which reflects the functional versatility of the gene products across different cellular compartments. Studies have demonstrated that certain proteins (e.g., receptor tyrosine kinases, G protein-coupled receptors) can receive signals at the cell membrane and translocate to the nucleus to directly regulate gene expression. For instance, the epidermal growth factor receptor (EGFR) translocates to the nucleus upon ligand binding and participates in transcriptional regulation [28]. The dual localization of *PkGLP1-2-6* and *PkGLP1-2-21* indicates that these proteins may act as multifunctional regulators involved in both signal transduction and transcriptional regulation. Their localization likely depends on the regulation of nuclear localization signals (NLS) and membrane localization signals (e.g., transmembrane domains or lipid modification sites), as well as cellular states or post-translational modifications (e.g., phosphorylation and ubiquitination) [29]. Upon viral infection, DNA damage, or oxidative stress, certain membrane proteins can translocate to the nucleus to participate in stress-responsive processes [30]. During the maturation of Korean pine somatic embryos, the application of exogenous ABA may activate *PkGLP1-2-6* and *PkGLP1-2-21*, thereby enabling these two proteins to participate in phytohormone signal transduction during the early phase of somatic embryogenesis. The elevated expression of *PkGLP1-2-6* and *PkGLP1-2-21* enables these proteins to respond to ABA signals, ultimately facilitating the formation of Korean pine somatic embryos.

### 3.4. The Role of PkGLP1-2-6 and PkGLP1-2-21 in Somatic Embryogenesis

Numerous genes associated with somatic embryogenesis have been reported in other plant species; however, functional investigations into Korean pine-related genes remain insufficiently in-depth. In this study, through the analysis of transcriptome data from Korean pine callus during somatic embryo maturation under different concentrations of ABA treatment, *PkGLP1-2-6* and *PkGLP1-2-21* were identified as core genes that play critical roles in ABA-mediated somatic embryo maturation. Using transgenic technology, we successfully obtained transgenic calli overexpressing *PkGLP1-2-6* and *PkGLP1-2-21*. When exogenous ABA at different concentrations was applied for somatic embryo maturation culture, the somatic embryo yield of the two transgenic calli was significantly increased. The results demonstrated that overexpression of *PkGLP1-2-6* and *PkGLP1-2-21* can effectively enhance the efficiency of somatic embryogenesis, increase the number of somatic embryos, and improve the quality of somatic embryos. Meanwhile, the phenotypic characteristics of each transgenic line exhibited significant differences from those of the empty vector control lines. These results confirm that the observed phenotypic alterations were specifically mediated by the overexpression of the target gene *PkGLP*, rather than arising from non-specific side effects associated with the genetic transformation process. This finding provides critical technical support for the genetic improvement and efficient propagation of Korean pine. Plant *GLP*s exhibit functional diversity; they are not only involved in the regulation of plant growth and development processes, but also play pivotal roles in mediating responses to both biotic and abiotic stresses. Numerous studies have confirmed that exogenous environmental stresses can significantly induce the transcriptional upregulation of plant *GLP* genes. This upregulation enhances *GLP* activity, promotes oxidative cross-linking of the cell wall, strengthens cell wall defense capabilities, mitigates ROS damage, and ultimately improves plant stress tolerance. In the study of abiotic stress responses, the expression of *ThGLP* genes in roots and leaves of *Tamarix ramosissima* was significantly induced by treatments with high salinity, drought, low temperature, ABA, and the heavy metal cadmium; this regulatory process is likely dependent on the ABA signal transduction pathway [31]. Previous transcriptome data indicated that exogenous ABA induced the up-regulation of *PkGLP1-2-6* and *PkGLP1-2-21*, leading to elevated endogenous ABA levels [13]. The ABA level accumulates rapidly and binds to ABA receptors to form a receptor-ligand complex, which subsequently provides a specific binding site for *PP2C*s. Binding of the complex to *PP2C*s inhibits the latter’s phosphatase activity, thereby suppressing the dephosphorylation of SnRK2s. *SnRK2s* are thereby released from the inhibitory state and further activate the transcription of a suite of downstream target genes, including those encoding transcription factors and channel proteins [32]. Endogenous ABA is synthesized via the carotenoid biosynthetic pathway; upon its production, the ABA receptor *PYR*/*PYL* binds to ABA, which triggers the formation of a *PYR*/*PYL*-ABA complex. This complex then interacts with *PP2C*, suppressing the phosphatase activity of *PP2C* and consequently relieving the inhibitory effect of *PP2C* on *SnRK2* kinase activity. Activated *SnRK2* kinases phosphorylate and thereby activate the downstream *ABF*s, a class of core transcription factors in the ABA signaling pathway. This activation, in turn, induces the expression of ABF-targeted downstream genes, which facilitates the accumulation of intracellular nutrients and ultimately modulates the maturation process of somatic embryos.

In this study, the contents of H_2_O_2_ and O_2_^−^ in the transgenic Korean pine calli were significantly reduced, indicating a decrease in ROS accumulation. This reduction alleviates oxidative damage, increases the yield and quality of somatic embryos, and lowers the proportion of abnormal embryos. This result is consistent with the findings from functional studies on the longan *GLP5* gene. In longan adventitious roots, the lignin content in *DlGLP1-5-1*-overexpressing lines was higher than that in WT lines, whereas the lignin content in knockout lines was reduced. This indicates that *GLP*s may affect lignin deposition by regulating H_2_O_2_ levels, thereby promoting root development [33]. The aforementioned studies have demonstrated that *GLP* overexpression exerts a positive effect on plant growth and development. This study further confirms that overexpression of *GLP* genes in Korean pine contributes to enhancing the efficiency of somatic embryogenesis.

## 4. Materials and Methods

### 4.1. Plant Materials

The plant materials were selected from the Korean pine seed orchard of Lushuihe Forestry Bureau, Jilin Province, China. The immature cones of Korean pine were collected in early July 2021, and a total of 5 cell lines were obtained by embryonic callus induction. By comparing the efficiency of somatic embryo maturation, the best line a-1 was selected as the main material of this study [13]. The maturation medium was: mLV added 1 g/L activated charcoal, 68 g/L sucrose (Sigma-Aldrich, Saint Louis, MO, USA), 12 g/L gellan gum (Sigma-Aldrich), 80 µmol/L ABA (Sigma-Aldrich), 0.5 g/L glutamine (Sigma-Aldrich) and 0.5 g/L acid hydrolyzed casein (Sigma-Aldrich), pH5.8 [14]. This cell line was subsequently subjected to proliferation culture. The proliferation medium was: mLV added 30 g/L sucrose (Sigma-Aldrich), 2 mg/L 2,4-D (Sigma-Aldrich), 0.5 mg/L 6-BA (Sigma-Aldrich), 0.5 g/L glutamine (Sigma-Aldrich) and 0.5 g/L acid hydrolyzed casein (Sigma-Aldrich), 4 g/L gellan gum (Sigma-Aldrich), pH5.8. Cell line a-1 was subjected to somatic embryo maturation culture with or without exogenous ABA supplementation. Subsequently, transcriptome sequencing analysis was performed on samples harvested at 0 d, 7 d, and 14 d of culture [13]. *Nicotiana benthamiana* was used as the experimental material and preserved in our laboratory. Tobacco seeds were rinsed with sterile water for 2 min; after the water was drained, the seeds were immersed in 30% (*v*/*v*) sodium hypochlorite solution for 15 min for surface sterilization. The seeds were rinsed with sterile water 4–5 times to remove residual sodium hypochlorite, then evenly spread on MS medium and cultured in a 25 °C constant-temperature tissue culture room under a 12 h light/12 h dark photoperiod. After 10 days of culture, the germinated seeds with elongated roots were transplanted into nutrient soil mixed at a ratio of soil to vermiculite = 1:1, and further grown under the same temperature and photoperiod conditions in the tissue culture room.

### 4.2. Construction of Weighted Gene Co-Expression Network

Based on our previously generated transcriptome dataset [13], a weighted gene co-expression network analysis (WGCNA) was performed using the WGCNA Shiny module integrated within the TBtools (v2. 210) software package. A normalized gene expression matrix was used as the input, which included transcriptomic profiles of 15 samples: these samples consisted of Korean pine somatic embryos subjected to exogenous treatment with 80 μmol/L ABA, and control embryos cultured without ABA for 0 d, 7 d, and 14 d during the maturation stage, with three biological replicates set for each sample group. To ensure data quality for network construction, genes were filtered based on the following criteria: the mean expression level in the preprocessed dataset was greater than 1, more than half of the samples had an expression level greater than 0, and the coefficient of variation (CV) exceeded 0.2. A soft threshold β value ranging from 1 to 30 was tested, and the optimal β was selected by calculating the corresponding correlation coefficients and gene average connectivity. Gene clustering and module division were conducted following the determination of the optimal soft threshold β. During module construction, the minimum number of genes per module was set to 30, and a module similarity merging threshold of 0.25 was adopted to merge highly similar modules and reduce redundancy. Subsequently, principal component analysis (PCA) was performed on the gene set within each co-expression module, where the first principal component (PC1) was defined as the module eigengene (ME), which represents the overall expression pattern of the corresponding module. To identify the co-expression modules specifically associated with ABA response, we calculated the correlation coefficient (r) between the module eigengene (ME) of each module and the ABA treatment phenotype, as well as the corresponding statistical significance (*p* value). A module was defined as an ABA response-specific module when the absolute value of the correlation coefficient |r| > 0.50 and the statistical significance *p* < 0.05. Subsequently, the identified modules meeting these criteria were selected for the construction of the weighted gene co-expression network.

### 4.3. Identification and Physicochemical Properties Analysis of PkGLP Gene Family Members

Based on the previously generated transcriptome data and the *Arabidopsis* database (TAIR) (https://www.arabidopsis.org/, accessed on 10 August 2025), the coding sequences (CDS) and corresponding amino acid sequences of *Arabidopsis GLP* genes were retrieved. Using the amino acid sequences as query probes, we performed one-way BLAST against the Korean pine genome via TBtools (v2. 210) for preliminary screening; this was followed by validation using two-way BLAST on the NCBI platform (https://www.ncbi.nlm.nih.gov/geo/, accessed on 10 August 2025). Finally, candidate *PkGLP* family members were obtained. The conserved domains of these candidate sequences were analyzed using the HMMER online tool (https://www.ebi.ac.uk/Tools/hmmer/search/phmmer, accessed on 10 August 2025). Subsequently, *PkGLP* members harboring complete Cupin-1 domains were screened out and systematically named based on the results of homologous alignment with *Arabidopsis thaliana*. The obtained *PkGLP* amino acid sequences were submitted to the ExPASy platform (http://au.expasy.org/) for analyzing their basic physicochemical properties, including the number of amino acids, isoelectric point, and molecular weight. The signal peptide sequences of the *PkGLP* family were predicted using SignalP 4.0 (http://www.cbs.dtu.dk/services/SignalP/, accessed on 12 August 2025), while subcellular localization analysis was conducted via WoLF PSORT (https://wolfpsort.hgc.jp/, accessed on 12 August 2025). Additionally, the tertiary structures of *PkGLP1-2-6* and *PkGLP1-2-21* were predicted with the SWISS-MODEL online platform.

### 4.4. Phylogenetic Tree and Collinearity Analysis of PkGLP Gene Family

Multiple sequence alignment was performed on the amino acid sequences of *PkGLP* and *GLP*s from *Arabidopsis thaliana*, rice, maize, and *Pinus tabulaeformis* using MEGA v7.0 software. A phylogenetic tree was constructed using the maximum likelihood method with the bootstrap value set to 1000 replicates. The resulting phylogenetic tree was then visualized and optimized on the iTOL online platform (https://itol.embl.de/upload.cgi, accessed on 13 August 2025). Based on the genome annotation information of Korean pine, the chromosomal localization map of *PkGLP* family members was plotted using TBtools (v2. 210). In addition, the gene collinearity relationship was analyzed via MCScanX, and the results were visualized using TBtools.

### 4.5. Gene Structure and Conservative Motif Analysis of PkGLP Gene Family

Based on the Korean pine genome annotation file, TBtools was used to analyze the gene structure characteristics of *PkGLP* family members. Conserved domain information was retrieved from the NCBI database, while conserved protein motifs were identified using the MEME online tool (http://meme-suite.org/, accessed on 13 August 2025), with the results subsequently visualized via TBtools.

### 4.6. Analysis of Cis-Acting Elements of PkGLP Promoter

Using the gene structure annotation file of the Korean pine genome, TBtools was employed to extract the 2000 bp sequence upstream of the translation initiation site of *PkGLP* family members, which was defined as the promoter region. The types and distribution patterns of cis-acting elements were systematically identified using the PlantCARE database (http://bioinformatics.psb.ugent.be/webtools/plantcare/html/, accessed on 15 August 2025), and the resultant analysis data were visualized via TBtools.

### 4.7. Expression Analysis of PkGLP Gene Under Abiotic Stress

In previous studies conducted by our laboratory, transcriptome sequencing was performed on Korean pine somatic embryo samples subjected to maturation culture for 0 d, 7 d and 14 d, with or without exogenous ABA (80 μmol/L) supplementation. Somatic embryo maturation culture was also carried out under the treatment of two different gellan gum concentrations (4 g/L and 12 g/L), and transcriptome sequencing analysis was subsequently performed on samples harvested at 0 h, 3 h and 12 h post-treatment. Based on the transcriptome data of Korean pine embryogenic calli under abiotic stress conditions (i.e., ABA and gellan gum treatments), we compared the 92 identified *GLP* family gene members against the transcriptome dataset, followed by screening for differentially expressed genes (DEGs) and subsequent analysis of the expression profiles of *PkGLP* genes. TBtools was employed to generate a visualized heat map of tissue-specific expression patterns, which was used to compare the *PkGLP* gene expression levels across different experimental samples. Seven *PkGLP* genes were selected for qPCR validation, with primer sequences detailed in Table S4. First, total RNA was first isolated from Korean pine somatic embryo samples (0 d, 7 d, 14 d of maturation culture; with or without exogenous ABA) and reverse-transcribed into cDNA, and three biological replicates were set up for each sample to ensure the reliability of the results. With *GAPDH* serving as the reference gene, the expression levels of the target *PkGLP* genes were detected via the SYBR Green I fluorescent dye method on an Analytik Jena EasyCycler PCR instrument (Germany). The relative expression levels of these genes were subsequently calculated using the 2^−ΔΔCt^ method.

### 4.8. Subcellular Localization of PkGLP1-2-6 and PkGLP1-2-21 Proteins in Tobacco

The full-length coding sequences (CDS) of *PkGLP1-2-6* and *PkGLP1-2-21* were amplified via PCR using a Bioer PCR instrument (Hangzhou, China), with Korean pine cDNA as the template and gene-specific primers *PkGLP1-2-6*-F/R and *PkGLP1-2-21*-F/R (primer sequences detailed in Table S5). These amplified full-length CDS fragments were subsequently inserted into the pFGC-eGFP vector to construct the corresponding recombinant plasmids. The agrobacterium suspensions containing the recombinant plasmids pFGC-*PkGLP1-2-6*-eGFP, pFGC-*PkGLP1-2-21*-eGFP, and the empty vector pFGC-eGFP were resuspended and then used for the transient infiltration of tobacco leaves. The infiltrated tobacco seedlings were placed in a culture room for dark incubation. After 48 h of incubation, the samples were observed using a high-resolution laser scanning confocal microscope (Zeiss LSM800, Shanghai, China) [34].

### 4.9. Stable Genetic Transformation of Embryogenic Callus of Korean Pine

The cloned *PkGLP1-2-6* and *PkGLP1-2-21* genes were directionally inserted into the linearized overexpression vector p1300-sGFP to generate the corresponding recombinant overexpression plasmids. The agrobacterium suspensions harboring the recombinant plasmids p1300-*PkGLP1-2-6*-sGFP, p1300-*PkGLP1-2-21*- sGFP and p1300-sGFP were resuspended. Subsequently, 2 g of Korean pine calli were immersed in 30–40 mL of the prepared infection solution for agroinfiltration. The agroinfiltration was carried out at 25 °C with a shaking speed of 120 rpm for 10 min. After infiltration, the excess infection solution was completely drained, and the calli were transferred to the co-culture medium supplemented with 100 μM acetosyringone (AS). The co-cultivation was performed in the dark at 25 ± 2 °C for 2 days. Subsequently, the calli were subjected to bacteriostasis treatment: they were rinsed thoroughly with a bacteriostatic solution containing 200 mg/L cefotaxime (cef) for three consecutive times, with each rinsing step lasting 5 min, and the bacteriostatic solution was discarded completely after each rinse. The treated calli were cultured on the recovery medium supplemented with 200 mg/L cefotaxime (cef) for 7 days to eliminate residual agrobacterium and facilitate the recovery of the explants. Subsequently, the calli were transferred to the selective medium containing 20 mg/L hygromycin (Hyg) for three successive rounds of screening, with each screening cycle lasting 21 days [35]. The resistant calli grown on the selective medium were subcultured for propagation. Total RNA was then extracted from the subcultured calli and reverse-transcribed into cDNA, which was used for molecular identification of the transgenic calli. Meanwhile, the GFP fluorescence signal of the p1300-sGFP vector in the transgenic calli was observed using a laser scanning confocal microscope (Zeiss LSM800, Shanghai, China). The wild-type material, p1300-sGFP, *PkGLP1-2-6*-OE-1, *PkGLP1-2-6*-OE-2 and *PkGLP1-2-21*-OE-1, *PkGLP1-2-21*-OE-2 transgenic callus were subjected to somatic embryo maturation culture with different concentrations of ABA (0, 80 μmol/L). When the mature culture of somatic embryos was 8 w, the number of somatic embryos formed by transgenic and wild-type callus of korean pine was counted.

### 4.10. Determination of Redox Substances in Wild-Type and Transgenic Callus and Somatic Embryos

The somatic embryos of 0.1 g WT callus (proliferation culture for 7 d) and its somatic embryo maturation culture for 8 w (0, 80 μmol/L ABA), and the somatic embryos of transgenic callus (proliferation culture for 7 d) and its somatic embryo maturation culture for 8 w (0, 80 μmol/L ABA) were weighed and repeated three times. The activities of three antioxidant enzymes, including SOD, POD, and CAT, as well as the contents of H_2_O_2_ and O_2_^−^ in the enzymatic reaction system were determined using commercial assay kits (Suzhou Keming, Suzhou, China).

### 4.11. Statistics

Microsoft Excel 2019 (Microsoft Corporation, Redmond, WA, USA) was used for data preprocessing. Statistical analyses of the somatic embryo number, gene expression levels, antioxidant enzyme (SOD, POD, CAT) activities, and reactive oxygen species (H_2_O_2_ and O_2_^−^) contents were performed using SPSS 21.0 (IBM, Ammonk, NY, USA) with one-way analysis of variance (ANOVA); differences were considered statistically significant at *p* < 0.05. GraphPad Prism 9.5.1 (GraphPad Software, San Diego, CA, USA) was employed to generate charts for visualizing the statistical results.

## 5. Conclusions

A total of 92 members of the *PkGLP* gene family, all containing the complete conserved Cupin-1 domain, were identified from the Korean pine genome. Phylogenetic analysis classified the *GLP* family members from Korean pine, *Arabidopsis thaliana*, *Oryza sativa*, *Zea mays*, and *Pinus tabulaeformis* into 8 distinct subfamilies. The *GLP* genes clustered in the same subfamily exhibit high sequence similarity. Moreover, some *PkGLP* members show continuously high expression levels across multiple developmental stages of Korean pine, which suggests that this gene family may play a regulatory role in the growth and development processes of Korean pine. Different *PkGLP* family genes may employ distinct molecular mechanisms to modulate the somatic embryogenesis process of Korean pine under exogenous ABA induction. Meanwhile, subcellular localization assays revealed that both *PkGLP1-2-6* and *PkGLP1-2-21* proteins were targeted to the cell membrane and nucleus. By utilizing the stable genetic transformation system of Korean pine, transgenic calli overexpressing *PkGLP1-2-6* and *PkGLP1-2-21* were successfully generated. Regardless of the presence or absence of exogenous ABA, the yield of somatic embryos derived from these transgenic calli was significantly higher than that of the WT control. ompared with the WT control, the SOD activity in the *PkGLP1-2-6* and *PkGLP1-2-21* overexpression lines was significantly increased, whereas the POD and CAT activities were reduced. Correspondingly, the contents of H_2_O_2_ and O_2_^−^ were significantly decreased in these overexpression lines. These results demonstrate that *PkGLP1-2-6* and *PkGLP1-2-21* play a pivotal regulatory role in the somatic embryogenesis of Korean pine by enhancing the ROS scavenging capacity of explants. Moreover, the overexpression of these two genes can significantly improve the yield of somatic embryos, highlighting their potential application value in the genetic improvement of Korean pine somatic embryogenesis efficiency.

## Data Availability

The datasets supporting the conclusions of this article are included within the article and its Supplementary Files.

## Supplementary Materials

The following supporting information can be downloaded at: https://www.mdpi.com/article/10.3390/plants15030476/s1, Figure S1: Core gene interaction network; Figure S2: Soft threshold screening; Figure S3: Correlation heat map between modules and traits; Figure S4: Conserved motif distribution and gene structure of *PkGLP* family members; Table S1: Physicochemical properties of proteins of *PkGLP* family members; Table S2: Expression analysis of *PkGLP* gene during the maturation of lower embryo under different concentrations of ABA; Table S3: Expression analysis of *PkGLP* gene during the maturation of lower body embryos under different concentrations of gellan gum; Table S4: Primer sequences; Table S5: The cloning primer sequences of *PkGLP1-2-6* and *PkGLP1-2-21*.
