# Supplementary material for: Genome Identification of GLP Family in Korean Pine and Study on the Function of GLP1-2-6/GLP1-2-21 in Somatic Embryo Maturation
Source: Plants (Basel). 2026 Feb 3;15(3):476. doi: 10.3390/plants15030476 (PMC12899398; doi:10.3390/plants15030476)
Supplement: Supplementary file 1 [file plants-15-00476-s001.zip › plants-4114671-SI.pdf]

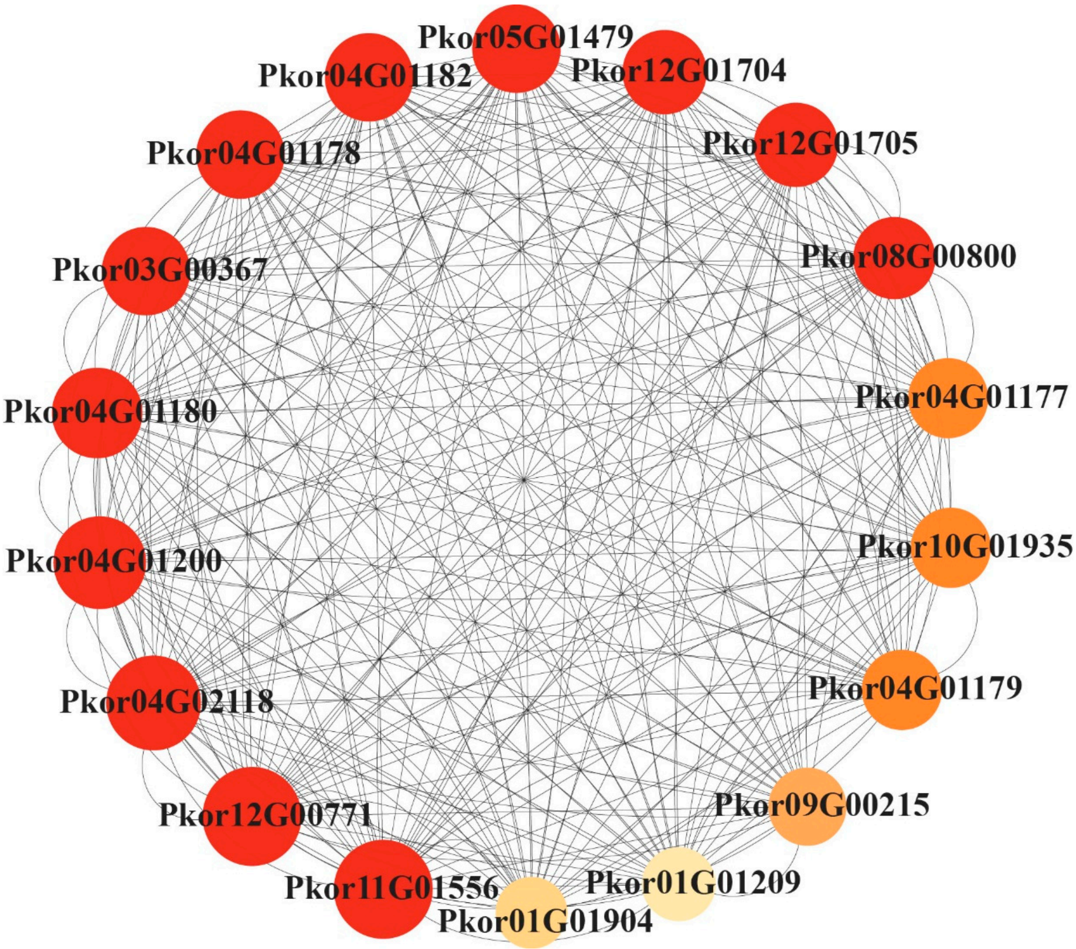

Figure S1 Core gene interaction network. Each node (depicted as a circle) represents a core gene. Both the size and color depth of the circles are positively correlated with the gene correla-tion degree — the larger the circle and the deeper the color, the higher the correlation.

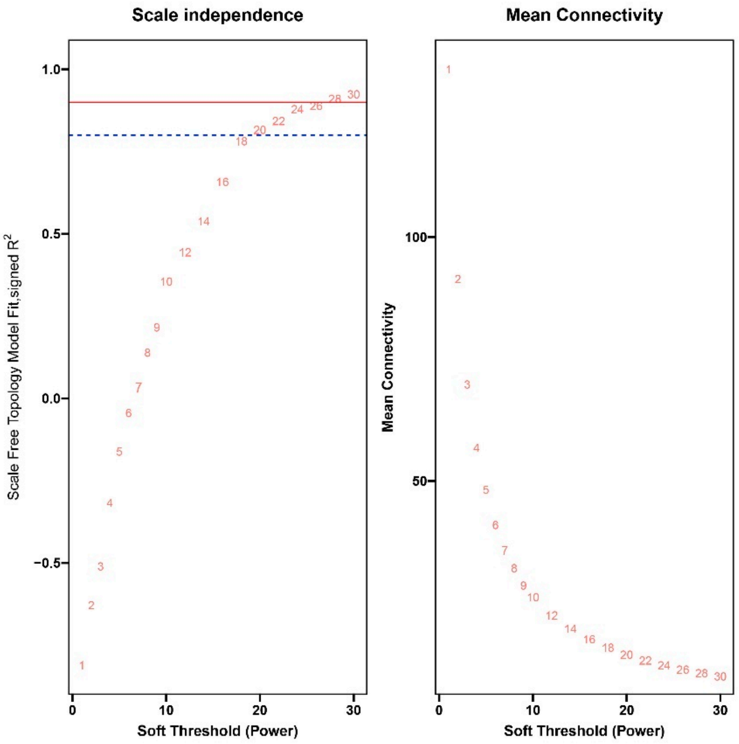

Figure S2 Soft threshold screening.

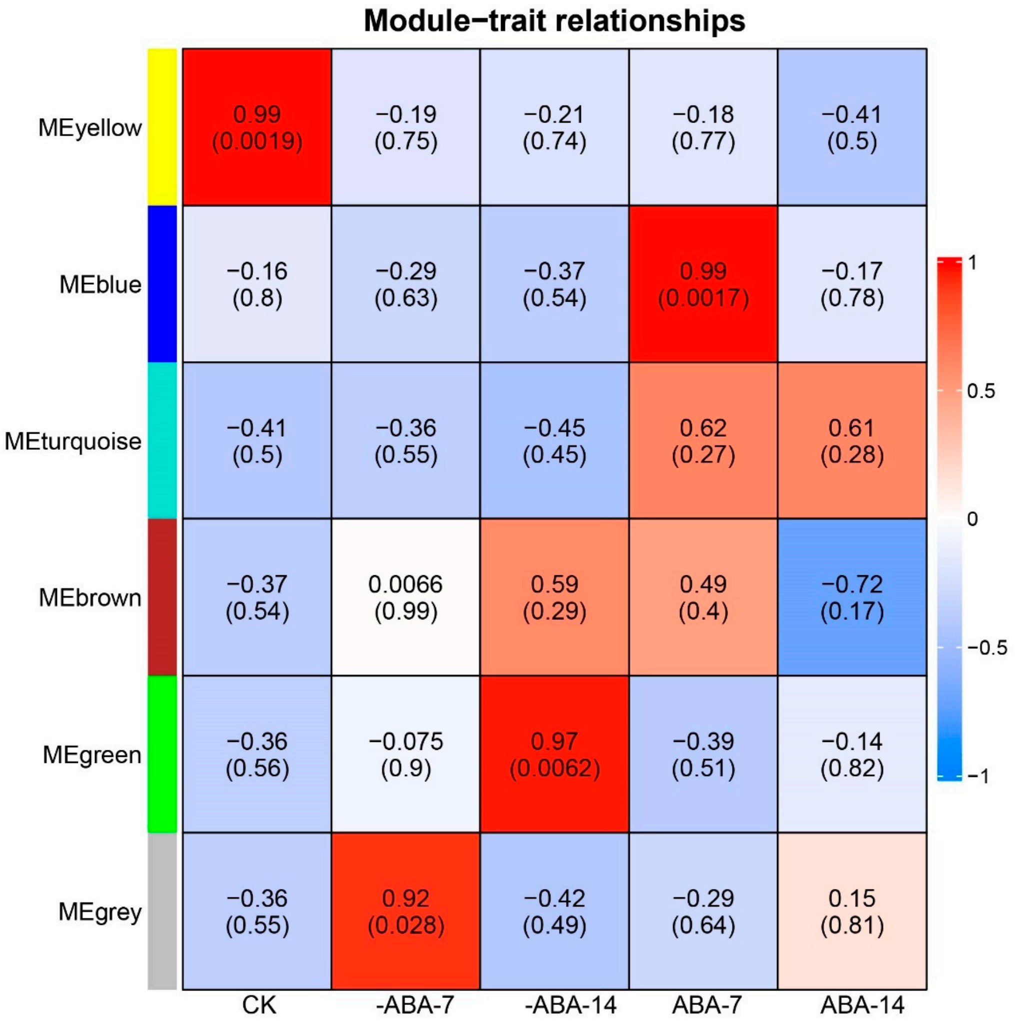

Figure S3 Correlation heat map between modules and traits. The abscissa shows the different time of mature culture of Korean pine somatic embryos under different concentrations of ABA, and the figures in the rectangular box are the correlation coefficient r and the corresponding P value (in parentheses). On the right is the color scale of heat map, indicating the change of correlation coefficient R.

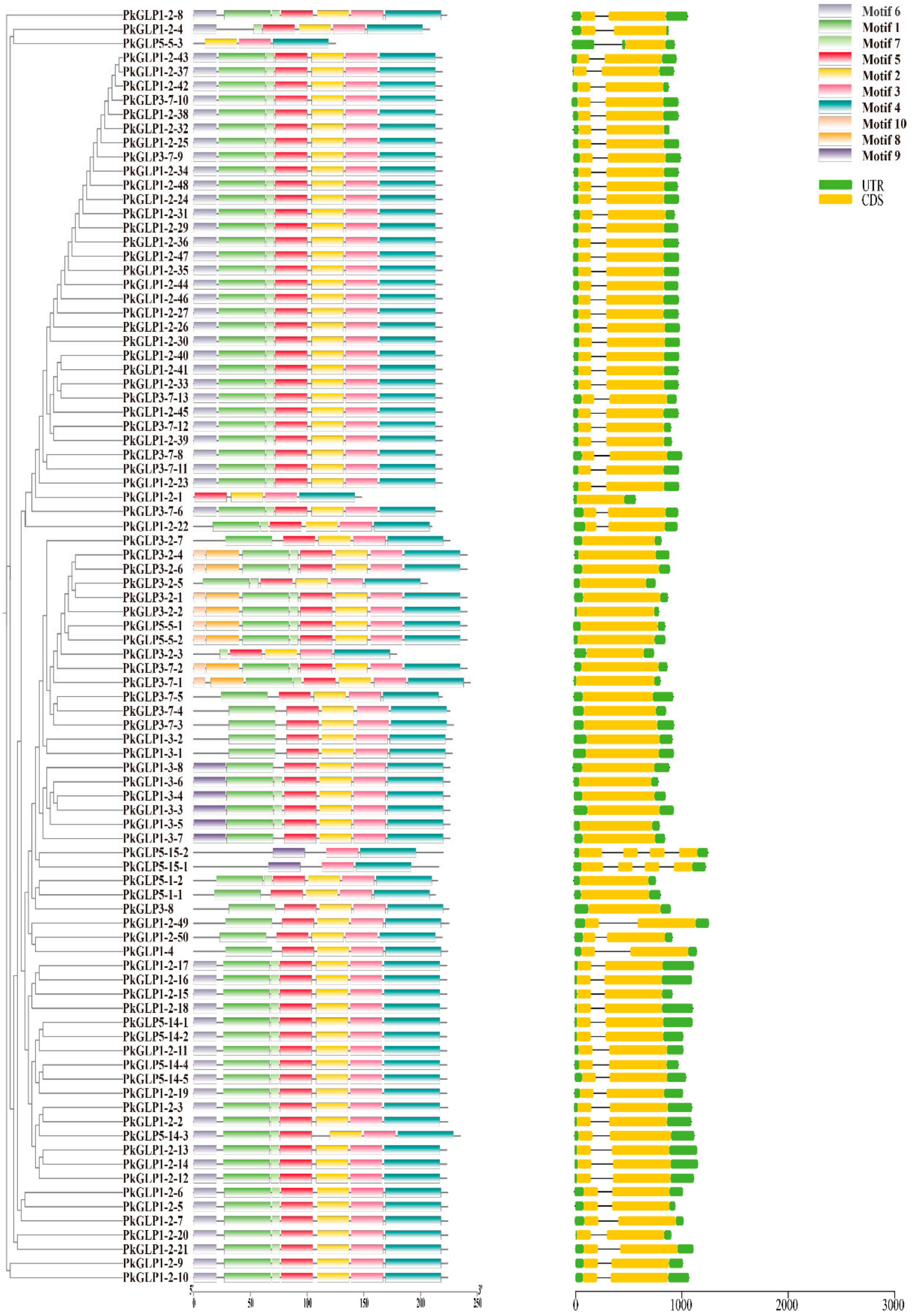

Figure S4 Conserved motif distribution and gene structure of *PkGLP* family members. Different colors represent different motif types. Distribution of conserved protein motifs identified through predictive analysis, with distinct colors representing different motif types. coding exons (yellow); non-coding flanking regions (green); intronic sequences (black lines).

Table S1 Physicochemical properties of proteins of *PkGLP* family members

Gene ID	Gene name	Cforresponding Arabidopsis name	Cforresponding Arabidopsis ID	Number of amino acid (aa)	Molecular weight	Theoretical pI	Instability index	Aliphatic index	Grand average of hydropathicity	α-helix	Extended strand	Random corn	Subcellular localization	Signal peptide
Pkor03G00261.1	*PkGLP1-2-1*	*AtGLP1-2*	*AT1G09560.1*	148	15690.21	9.1	20.29	105.47	0.339	18.92%	32.43%	48.65%	chlo	No
Pkor04G01184.1	*PkGLP1-2-10*	*AtGLP1-2*	*AT1G09560.1*	224	23720.6	6.5	22.57	109.2	0.397	24.55%	27.68%	47.77%	E.R.	Yes
Pkor04G01186.1	*PkGLP1-2-11*	*AtGLP1-2*	*AT1G09560.1*	223	23685.23	6.4	27.24	104.93	0.293	18.92%	32.43%	48.43%	vacu	Yes
Pkor04G01190.1	*PkGLP1-2-12*	*AtGLP1-2*	*AT1G09560.1*	223	23601.4	6.9	22.92	109.24	0.373	24.22%	27.80%	47.98%	plas	Yes
Pkor04G01191.1	*PkGLP1-2-13*	*AtGLP1-2*	*AT1G09560.1*	223	23609.39	5.85	22.73	110.13	0.384	23.32%	27.35%	49.33%	vacu	Yes
Pkor04G01192.1	*PkGLP1-2-14*	*AtGLP1-2*	*AT1G09560.1*	223	23699.59	6.9	23.32	111.43	0.379	24.22%	27.80%	47.98%	plas	Yes
Pkor04G01194.1	*PkGLP1-2-15*	*AtGLP1-2*	*AT1G09560.1*	223	23897.75	6.26	27.6	108.83	0.347	24.22%	26.91%	48.88%	vacu	Yes
Pkor04G01195.1	*PkGLP1-2-16*	*AtGLP1-2*	*AT1G09560.1*	223	23897.75	6.26	27.6	108.83	0.347	24.22%	26.91%	48.88%	vacu	Yes
Pkor04G01196.1	*PkGLP1-2-17*	*AtGLP1-2*	*AT1G09560.1*	223	23897.75	6.26	27.6	108.83	0.347	24.22%	26.91%	48.88%	vacu	Yes
Pkor04G01197.1	*PkGLP1-2-18*	*AtGLP1-2*	*AT1G09560.1*	223	23897.75	6.26	27.6	108.83	0.347	24.22%	26.91%	48.88%	vacu	Yes
Pkor04G01198.1	*PkGLP1-2-19*	*AtGLP1-2*	*AT1G09560.1*	223	23748.35	4.87	33.04	106.68	0.392	24.66%	27.35%	47.98%	plas	Yes
Pkor04G01077.1	*PkGLP1-2-2*	*AtGLP1-2*	*AT1G09560.1*	224	23801.6	6.39	24.52	112.68	0.412	23.66%	26.79%	49.55%	plas	Yes
Pkor04G01199.1	*PkGLP1-2-20*	*AtGLP1-2*	*AT1G09560.1*	224	23629.44	6.5	26.87	108.3	0.375	24.55%	27.68%	47.77%	E.R.	Yes
Pkor04G01200.1	*PkGLP1-2-21*	*AtGLP1-2*	*AT1G09560.1*	224	23699.49	6.17	27.06	105.71	0.387	22.32%	26.79%	50.89%	chlo	Yes
Pkor04G01342.1	*PkGLP1-2-22*	*AtGLP1-2*	*AT1G09560.1*	210	22080.36	5.72	20.31	95.71	0.301	22.38%	29.05%	48.57%	chlo	Yes
Pkor04G02669.1	*PkGLP1-2-23*	*AtGLP1-2*	*AT1G09560.1*	219	22935.56	6.04	20.9	106.03	0.414	23.29%	27.85%	48.86%	extr	Yes
Pkor04G02670.1	*PkGLP1-2-24*	*AtGLP1-2*	*AT1G09560.1*	219	22921.53	6.04	20.9	105.57	0.413	24.20%	27.40%	48.40%	extr	Yes
Pkor04G02671.1	*PkGLP1-2-25*	*AtGLP1-2*	*AT1G09560.1*	219	22921.53	6.04	20.9	105.57	0.413	24.20%	27.40%	48.40%	extr	Yes
Pkor04G02672.1	*PkGLP1-2-26*	*AtGLP1-2*	*AT1G09560.1*	219	22921.53	6.04	20.9	105.57	0.413	24.20%	27.40%	48.40%	extr	Yes
Pkor04G02673.1	*PkGLP1-2-27*	*AtGLP1-2*	*AT1G09560.1*	219	22921.53	6.04	20.9	105.57	0.413	24.20%	27.40%	48.40%	extr	Yes
Pkor04G02674.1	*PkGLP1-2-28*	*AtGLP1-2*	*AT1G09560.1*	219	22921.53	6.04	20.9	105.57	0.413	24.20%	27.40%	48.40%	extr	Yes
Pkor04G02675.1	*PkGLP1-2-29*	*AtGLP1-2*	*AT1G09560.1*	219	22921.53	6.04	20.9	105.57	0.413	24.20%	27.40%	48.40%	extr	Yes

Table S1 Physicochemical properties of proteins of *PkGLP* family members(continued)

Gene ID	Gene name	Cforresponding Arabidopsis name	Cforresponding Arabidopsis ID	Number of amino acid (aa)	Molecular weight	Theoretical pI	Instability index	Aliphatic index	Grand average of hydropathicity	α-helix	Extended strand	Random corn	Subcellular localization
Pkor04G01078.1	*PkGLP1-2-3*	*AtGLP1-2*	*AT1G09560.1*	224	23801.6	6.39	24.52	112.68	0.412	23.66%	26.79%	49.55%	plas
Pkor04G02676.1	*PkGLP1-2-30*	*AtGLP1-2*	*AT1G09560.1*	219	22921.53	6.04	20.9	105.57	0.413	24.20%	27.40%	48.40%	extr
Pkor04G02677.1	*PkGLP1-2-31*	*AtGLP1-2*	*AT1G09560.1*	219	22921.53	6.04	20.9	105.57	0.413	24.20%	27.40%	48.40%	extr
Pkor04G02678.1	*PkGLP1-2-32*	*AtGLP1-2*	*AT1G09560.1*	219	22921.53	6.04	20.9	105.57	0.413	24.20%	27.40%	48.40%	extr
Pkor04G02679.1	*PkGLP1-2-33*	*AtGLP1-2*	*AT1G09560.1*	219	22949.58	6.04	20.9	106.44	0.424	23.74%	27.85%	48.40%	extr
Pkor04G02680.1	*PkGLP1-2-34*	*AtGLP1-2*	*AT1G09560.1*	219	22921.53	6.04	20.9	105.57	0.413	24.20%	27.40%	48.40%	extr
Pkor04G02681.1	*PkGLP1-2-35*	*AtGLP1-2*	*AT1G09560.1*	219	22921.53	6.04	20.9	105.57	0.413	24.20%	27.40%	48.40%	extr
Pkor04G02682.1	*PkGLP1-2-36*	*AtGLP1-2*	*AT1G09560.1*	219	22921.53	6.04	20.9	105.57	0.413	24.20%	27.40%	48.40%	extr
Pkor04G02684.1	*PkGLP1-2-37*	*AtGLP1-2*	*AT1G09560.1*	219	22921.53	6.04	20.9	105.57	0.413	24.20%	27.40%	48.40%	extr
Pkor04G02685.1	*PkGLP1-2-38*	*AtGLP1-2*	*AT1G09560.1*	219	22921.53	6.04	20.9	105.57	0.413	24.20%	27.40%	48.40%	extr
Pkor04G02686.1	*PkGLP1-2-39*	*AtGLP1-2*	*AT1G09560.1*	219	22905.49	6.04	22.66	103.79	0.388	23.29%	27.40%	49.32%	extr
Pkor04G01178.1	*PkGLP1-2-4*	*AtGLP1-2*	*AT1G09560.1*	208	22180.74	6.17	24.31	109.62	0.341	25.96%	28.37%	45.67%	E.R.
Pkor04G02687.1	*PkGLP1-2-60*	*AtGLP1-2*	*AT1G09560.1*	219	22908.53	6.04	21.29	105.57	0.426	21.92%	26.94%	51.14%	extr
Pkor04G02689.1	*PkGLP1-2-61*	*AtGLP1-2*	*AT1G09560.1*	219	22969.62	6.4	21.98	105.57	0.423	23.29%	27.85%	48.86%	extr
Pkor04G02690.1	*PkGLP1-2-62*	*AtGLP1-2*	*AT1G09560.1*	219	22921.53	6.04	20.9	105.57	0.413	24.20%	27.40%	48.40%	extr
Pkor04G02691.1	*PkGLP1-2-63*	*AtGLP1-2*	*AT1G09560.1*	219	22921.53	6.04	20.9	105.57	0.413	24.20%	27.40%	48.40%	extr
Pkor04G02692.1	*PkGLP1-2-64*	*AtGLP1-2*	*AT1G09560.1*	219	22921.53	6.04	20.9	105.57	0.413	24.20%	27.40%	48.40%	extr
Pkor04G02693.1	*PkGLP1-2-65*	*AtGLP1-2*	*AT1G09560.1*	219	22921.53	6.04	20.9	105.57	0.413	24.20%	27.40%	48.40%	extr
Pkor04G02696.1	*PkGLP1-2-66*	*AtGLP1-2*	*AT1G09560.1*	219	22921.53	6.04	20.9	105.57	0.413	24.20%	27.40%	48.40%	extr
Pkor04G02697.1	*PkGLP1-2-67*	*AtGLP1-2*	*AT1G09560.1*	219	22921.53	6.04	20.9	105.57	0.413	24.20%	27.40%	48.40%	extr
Pkor04G02698.1	*PkGLP1-2-68*	*AtGLP1-2*	*AT1G09560.1*	219	22921.53	6.04	20.9	105.57	0.413	24.20%	27.40%	48.40%	extr
Pkor06G01230.1	*PkGLP1-2-69*	*AtGLP1-2*	*AT1G09560.1*	225	23988.46	6.4	24.89	94	0.248	22.22%	27.11%	50.67%	plas

Table S1 Physicochemical properties of proteins of *PkGLP* family members(continued)

**Gene ID**	**Gene name**	**Cforresponding Arabidopsis name**	**Cforresponding Arabidopsis ID**	**Number of amino acid (aa)**	**Molecular weight**	**Theoretical pI**	**Instability index**	**Aliphatic index**	**Grand average of hydropathicity**	**α-helix**	**Extended strand**	**Random corn**	**Subcellular localization**
Pkor04G01179.1	*PkGLP1-2-5*	*AtGLP1-2*	*AT1G09560.1*	224	23672.5	6.04	23.81	108.75	0.409	25.00%	27.23%	47.77%	E.R.
Pkor06G01233.1	*PkGLP1-2-50*	*AtGLP1-2*	*AT1G09560.1*	219	23360.93	6.28	31.91	96.16	0.284	23.74%	27.40%	48.86%	extr
Pkor04G01180.1	*PkGLP1-2-6*	*AtGLP1-2*	*AT1G09560.1*	224	23672.5	6.04	23.81	108.75	0.409	25.00%	27.23%	47.77%	E.R.
Pkor04G01181.1	*PkGLP1-2-7*	*AtGLP1-2*	*AT1G09560.1*	224	23672.5	6.04	23.81	108.75	0.409	25.00%	27.23%	47.77%	E.R.
Pkor04G01182.1	*PkGLP1-2-8*	*AtGLP1-2*	*AT1G09560.1*	223	23633.52	6.5	21.76	109.69	0.402	24.66%	27.35%	47.98%	E.R.
Pkor04G01183.1	*PkGLP1-2-9*	*AtGLP1-2*	*AT1G09560.1*	224	23720.6	6.5	22.57	109.2	0.397	24.55%	27.68%	47.77%	E.R.
Pkor01G01488.1	*PkGLP1-3-1*	*AtGLP1-3*	*AT1G10460.1*	228	24104	8.88	26.09	93.25	0.363	25.00%	26.32%	48.68%	extr
Pkor01G01489.1	*PkGLP1-3-2*	*AtGLP1-3*	*AT1G10460.1*	228	24186.41	9.59	29.34	98.82	0.434	23.68%	27.19%	49.12%	extr
Pkor08G02058.1	*PkGLP1-3-3*	*AtGLP1-3*	*AT1G10460.1*	226	24111.04	9.44	26.11	97.96	0.259	23.45%	26.55%	50.00%	chlo
Pkor08G02059.1	*PkGLP1-3-4*	*AtGLP1-3*	*AT1G10460.1*	226	24230.26	9.55	28.79	97.96	0.265	24.34%	27.43%	48.23%	nucl
Pkor08G02061.1	*PkGLP1-3-5*	*AtGLP1-3*	*AT1G10460.1*	226	24103.11	9.05	26.12	100.13	0.313	23.01%	27.88%	49.12%	E.R.
Pkor08G02062.1	*PkGLP1-3-6*	*AtGLP1-3*	*AT1G10460.1*	226	24149.19	9.44	26.47	100.58	0.272	22.57%	26.99%	50.44%	nucl
Pkor08G02063.1	*PkGLP1-3-7*	*AtGLP1-3*	*AT1G10460.1*	226	23870.52	8.46	24.94	94.07	0.265	24.78%	26.55%	48.67%	cyt
Pkor08G02064.1	*PkGLP1-3-8*	*AtGLP1-3*	*AT1G10460.1*	226	24114.16	9.14	26.77	97.48	0.327	23.01%	27.88%	49.12%	vacu
Pkor06G01229.1	*PkGLP1-4*	*AtGLP1-4*	*AT1G18970.1*	224	23920.73	8.44	28.15	90.89	0.098	22.77%	28.12%	49.11%	xtr:
Pkor01G01476.1	*PkGLP3-2-1*	*AtGLP3-2*	*AT3G04170.1*	241	25767.93	5.82	44.48	102.82	0.357	24.48%	25.73%	49.79%	E.R.
Pkor01G01477.1	*PkGLP3-2-2*	*AtGLP3-2*	*AT3G04170.1*	241	25765.92	5.82	43.45	101.2	0.332	23.24%	25.73%	51.04%	E.R.
Pkor01G01479.1	*PkGLP3-2-3*	*AtGLP3-2*	*AT3G04170.1*	179	18875.76	6.96	42.14	100.84	0.149	15.64%	30.73%	53.63%	chlo
Pkor01G01480.1	*PkGLP3-2-4*	*AtGLP3-2*	*AT3G04170.1*	241	25793.97	5.82	43.45	101.99	0.342	24.48%	27.39%	48.13%	E.R.
Pkor01G01481.1	*PkGLP3-2-5*	*AtGLP3-2*	*AT3G04170.1*	206	21735.88	5.67	43.17	96.17	0.103	14.56%	33.01%	52.43%	cysk
Pkor01G01483.1	*PkGLP3-2-6*	*AtGLP3-2*	*AT3G04170.1*	241	25808	5.82	43.45	102.41	0.341	23.24%	24.90%	51.87%	E.R.
Pkor01G01496.1	*PkGLP3-2-7*	*AtGLP3-2*	*AT3G04170.1*	226	23430.11	8.37	28.74	101.5	0.329	23.01%	27.43%	49.56%	cyto

Table S1 Physicochemical properties of proteins of *PkGLP* family members(continued)

Gene ID	Gene name	Cforresponding Arabidopsis name	Cforresponding Arabidopsis ID	Number of amino acid (aa)	Molecular weight	Theoretical pI	Instability index	Aliphatic index	Grand average of hydropathicity	α-helix	Extended strand	Random corn	Subcellular localization
Pkor01G01474.1	*PkGLP3-7-1*	*AtGLP3-7*	*AT3G05950.1*	244	26095.27	5.68	48.74	101.15	0.302	29.92%	25.00%	45.08%	mito
Pkor04G02667.1	*PkGLP3-7-10*	*AtGLP3-7*	*AT3G05950.1*	219	22921.53	6.04	20.9	105.57	0.413	24.20%	27.40%	48.40%	extr
Pkor04G02668.1	*PkGLP3-7-11*	*AtGLP3-7*	*AT3G05950.1*	219	22951.56	6.04	20.21	105.11	0.401	22.83%	27.40%	49.77%	extr
Pkor04G02688.1	*PkGLP3-7-12*	*AtGLP3-7*	*AT3G05950.1*	219	22922.47	5.45	22.78	105.57	0.415	22.83%	28.31%	48.86%	extr
Pkor04G02694.1	*PkGLP3-7-13*	*AtGLP3-7*	*AT3G05950.1*	219	22965.63	6.04	19.14	108.22	0.448	22.83%	27.40%	49.77%	extr
Pkor01G01484.1	*PkGLP3-7-2*	*AtGLP3-7*	*AT3G05950.1*	241	25923.12	5.82	47.2	102.82	0.315	24.48%	26.56%	48.96%	E.R.
Pkor01G01486.1	*PkGLP3-7-3*	*AtGLP3-7*	*AT3G05950.1*	229	24466.23	6.9	35.39	96.99	0.309	22.71%	27.07%	50.22%	chlo
Pkor01G01487.1	*PkGLP3-7-4*	*AtGLP3-7*	*AT3G05950.1*	226	24316.22	8.49	49.2	92.26	0.239	23.45%	26.55%	50.00%	cyto
Pkor03G02030.1	*PkGLP3-7-5*	*AtGLP3-7*	*AT3G05950.1*	219	23647.36	7.77	47.22	92.51	0.208	23.29%	27.85%	48.86%	vacu
Pkor04G01341.1	*PkGLP3-7-6*	*AtGLP3-7*	*AT3G05950.1*	219	22941.45	5.72	19.88	102.47	0.38	22.83%	26.48%	50.68%	extr
Pkor04G01343.1	*PkGLP3-7-7*	*AtGLP3-7*	*AT3G05950.1*	219	22969.5	5.72	19.88	103.33	0.391	21.46%	27.85%	50.68%	extr
Pkor04G01355.1	*PkGLP3-7-8*	*AtGLP3-7*	*AT3G05950.1*	219	22980.6	6.4	18.8	105.57	0.4	23.29%	27.40%	49.32%	extr
Pkor04G01356.1	*PkGLP3-7-9*	*AtGLP3-7*	*AT3G05950.1*	219	22921.53	6.04	20.9	105.57	0.413	24.20%	27.40%	48.40%	extr
Pkor12G00734.1	*PkGLP3-8*	*AtGLP3-8*	*AT3G10080.1*	225	23979.64	7.72	19.23	93.47	0.153	24.89%	27.11%	48.00%	xtr:
Pkor06G01558.1	*PkGLP5-1-1*	*AtGLP5-1*	*AT5G20630.1*	213	22370.77	5.49	42.33	103.99	0.472	21.60%	28.17%	50.23%	extr
Pkor06G01559.1	*PkGLP5-1-2*	*AtGLP5-1*	*AT5G20630.1*	215	22409.87	7.71	45.33	102.56	0.498	23.72%	28.37%	47.91%	extr
Pkor04G01187.1	*PkGLP5-14-1*	*AtGLP5-14*	*AT5G39190.1*	223	23778.35	5.72	25.25	108.39	0.313	25.11%	26.91%	47.98%	plas
Pkor04G01188.1	*PkGLP5-14-2*	*AtGLP5-14*	*AT5G39190.1*	223	23778.35	5.72	25.25	107.94	0.316	25.11%	27.35%	47.53%	plas
Pkor04G01189.1	*PkGLP5-14-3*	*AtGLP5-14*	*AT5G39190.1*	235	25249.26	8.67	24.38	104.94	0.251	21.70%	30.64%	47.66%	plas
Pkor04G01202.1	*PkGLP5-14-4*	*AtGLP5-14*	*AT5G39190.1*	223	23674.28	7.81	27.51	101.03	0.295	22.87%	28.25%	48.88%	plas
Pkor04G01203.1	*PkGLP5-14-5*	*AtGLP5-14*	*AT5G39190.1*	223	23730.51	9.44	27.65	103.63	0.261	25.11%	26.91%	47.98%	plas
Pkor02G00787.1	*PkGLP5-15-1*	*AtGLP5-15*	*AT5G61750.1*	216	22906.03	7.85	22.16	93.38	0.068	20.83%	24.54%	54.63%	extr

Table S1 Physicochemical properties of proteins of *PkGLP* family members(continued)

Gene ID	Gene name	Cforresponding Arabidopsis name	Cforresponding Arabidopsis ID	Number of amino acid (aa)	Molecular weight	Theoretical pI	Instability index	Aliphatic index	Grand average of hydropathicity	α-helix	Extended strand	Random corn	Subcellular localization
Pkor02G00794.1	*PkGLP5-15-2*	*AtGLP5-15*	*AT5G61750.1*	220	23314.59	8.66	27.54	91.68	0.07	23.18%	25.45%	51.36%	vacu
Pkor01G01478.1	*PkGLP5-5-1*	*AtGLP5-5*	*AT5G38940.1*	241	25751.85	5.61	42.82	101.2	0.334	24.90%	26.14%	48.96%	E.R.
Pkor01G01482.1	*PkGLP5-5-2*	*AtGLP5-5*	*AT5G38940.1*	241	25751.85	5.61	42.82	101.2	0.334	24.90%	26.14%	48.96%	E.R.
Pkor04G01177.1	*PkGLP5-5-3*	*AtGLP5-5*	*AT5G38940.1*	125	13389.6	9.6	28.73	109.84	0.308	23.20%	33.60%	43.20%	cyto

Table S2 Expression analysis of *PkGLP* gene during the maturation of lower embryo under different concentrations of ABA

Gene ID	Gene ID	ck	-ABA-7	-ABA-14	ABA-7	ABA-14
Pkor01G01474	*PkGLP3-7-1*	0.62	4.636667	3.573333	3.753333	1.02
Pkor01G01476	*PkGLP3-2-1*	0	0.09	0	0.07	0.07
Pkor01G01477	*PkGLP3-2-2*	0.226667	2.02	1.533333	5.52	1.453333
Pkor01G01478	*PkGLP5-5-1*	0.066667	0	0.04	0	0
Pkor01G01480	*PkGLP3-2-4*	0.023333	0	0	0	0
Pkor01G01481	*PkGLP3-2-5*	0	0	0	0	0.076667
Pkor01G01482	*PkGLP5-5-2*	0.066667	0	0.04	0	0
Pkor01G01483	*PkGLP3-2-6*	0.023333	0	0	0	0
Pkor01G01484	*PkGLP3-7-2*	0	0	0	0.086667	0
Pkor01G01487	*PkGLP3-7-4*	5.87	19.92667	3.066667	24.21333	20.23667
Pkor01G01488	*PkGLP1-3-1*	1991.183	1536.177	1408.087	1939.67	1506.18
Pkor01G01489	*PkGLP1-3-2*	110.5533	99.2	77.98667	125.2433	58.94667
Pkor01G01496	*PkGLP3-2-7*	0	0	0	0.076667	0
Pkor02G00787	*PkGLP5-15-1*	240.74	98.51	65.9	147.2233	86.43
Pkor02G00794	*PkGLP5-15-2*	325.9867	152.57	144.05	213.1	139.77
Pkor03G02030	*PkGLP3-7-5*	0.426667	0.133333	0.066667	0.55	0.27
Pkor04G01077	*PkGLP1-2-2*	1.076667	0	0.066667	0	0.096667
Pkor04G01078	*PkGLP1-2-3*	1.076667	0	0.066667	0	0.096667
Pkor04G01177	*PkGLP5-5-3*	0.436667	6.33	3.283333	27.00333	4.316667
Pkor04G01178	*PkGLP1-2-4*	0.08	4.24	2.423333	26.95333	4.273333
Pkor04G01179	*PkGLP1-2-5*	1.496667	21.03	12.13333	93.56	16.95333
Pkor04G01180	*PkGLP1-2-6*	0.25	2.036667	1.56	10.41333	1.763333
Pkor04G01181	*PkGLP1-2-7*	3.733333	43.56333	24.32333	168.9	30.62667
Pkor04G01182	*PkGLP1-2-8*	0.523333	8.346667	3.166667	53.03333	5.726667
Pkor04G01183	*PkGLP1-2-9*	0.336667	5.813333	3.51	22.50667	4.55
Pkor04G01184	*PkGLP1-2-10*	0.336667	5.813333	3.51	22.50667	4.55
Pkor04G01186	*PkGLP1-2-11*	0.56	1.77	1.106667	0.526667	0.52
Pkor04G01187	*PkGLP5-14-1*	0.226667	0.293333	0.716667	0.953333	0.233333
Pkor04G01188	*PkGLP5-14-2*	0.066667	0.223333	0.286667	0.366667	0.42
Pkor04G01189	*PkGLP5-14-3*	0.186667	0	0	0.06	0
Pkor04G01190	*PkGLP1-2-12*	0	0.063333	0	0	0
Pkor04G01191	*PkGLP1-2-13*	0.07	0.196667	0	0.066667	0.13
Pkor04G01192	*PkGLP1-2-14*	0.203333	0	0	0	0
Pkor04G01194	*PkGLP1-2-15*	0.88	0.386667	0.26	1.66	0.67
Pkor04G01195	*PkGLP1-2-16*	0	0	0.526667	0	0.156667
Pkor04G01197	*PkGLP1-2-18*	0.346667	0.53	0.206667	0.893333	0.156667
Pkor04G01198	*PkGLP1-2-19*	0	0.063333	0.066667	0.066667	0.133333
Pkor04G01199	*PkGLP1-2-20*	0.256667	5.183333	2.726667	18.87667	3.203333
Pkor04G01200	*PkGLP1-2-21*	0.313333	7.476667	2.423333	39.06	5.236667
Pkor04G01202	*PkGLP5-14-4*	0	0	0.066667	0	0
Pkor04G01203	*PkGLP5-14-5*	0	0.586667	0.326667	0.2	0.196667

Table S2 Expression analysis of *PkGLP* gene during the maturation of lower embryo under different concentrations of ABA(continued)

Gene ID	Gene ID	ck	-ABA-7	-ABA-14	ABA-7	ABA-14
Pkor01G01476	*PkGLP3-2-1*	0	0.09	0	0.07	0.07
Pkor01G01477	*PkGLP3-2-2*	0.226667	2.02	1.533333	5.52	1.453333
Pkor04G01341	*PkGLP3-7-6*	0.073333	0.23	0.07	0	0
Pkor04G01342	*PkGLP1-2-22*	0	0.103333	0	0	0
Pkor04G01343	*PkGLP3-7-7*	0.1	0.513333	0.193333	0.803333	0.19
Pkor04G01355	*PkGLP3-7-8*	0.033333	0	0.166667	0	0.07
Pkor04G01356	*PkGLP3-7-9*	0.106667	2.51	0.44	6.953333	0.14
Pkor06G01229	*PkGLP1-4*	1.976667	4.47	2.206667	10.26	5.163333
Pkor06G01230	*PkGLP1-2-49*	2.366667	2.563333	1.486667	2.126667	0.713333
Pkor06G01233	*PkGLP1-2-50*	0.216667	2.23	0.883333	9.373333	0.87
Pkor06G01559	*PkGLP5-1-2*	13.87	8.636667	3.59	11.18333	5.203333
Pkor08G02058	*PkGLP1-3-3*	6.25	19.41	20.89	5.32	11.02
Pkor08G02059	*PkGLP1-3-4*	17.89333	27.43333	26.23667	8.666667	18.49667
Pkor08G02061	*PkGLP1-3-5*	2.753333	3.24	3.653333	2.586667	2.99
Pkor08G02062	*PkGLP1-3-6*	3.833333	5.48	6.083333	2.69	5.023333
Pkor08G02063	*PkGLP1-3-7*	0.203333	0.063333	0	0.063333	0.066667
Pkor08G02064	*PkGLP1-3-8*	0.066667	0	0	0.066667	0.256667
Pkor12G00734	*PkGLP3-8*	211.3	65.58333	71.85	86.37	109.2133

Table S3 Expression analysis of *PkGLP* gene during the maturation of lower body embryos under different concentrations of gellan gum

Gene ID	Gene ID	ck	4g-3h	12g-3h	4g-6h	12g-6h	4g-12h	12g-12h	4g-24h	12g-24h
Pkor01G01474	*PkGLP3-7-1*	*0.55033333*	0.82166667	0.191	0.08833333	0.07266667	0.376	0.12866667	0.131	0.071
Pkor01G01484	*PkGLP3-7-2*	*0*	0.027	0.028	0.028	0.02733333	0.01333333	0.01333333	0	0.01333333
Pkor01G01486	*PkGLP3-7-3*	*0.01266667*	0	0.03833333	0.01233333	0.01266667	0.02533333	0.02433333	0.02533333	0.063
Pkor01G01487	*PkGLP3-7-4*	*0.52966667*	18.9266667	11.3503333	8.01666667	5.56033333	24.5763333	15.733	6.94866667	4.65033333
Pkor01G01488	*PkGLP1-3-1*	*400.124667*	455.185667	320.348333	198.12	216.892667	447.879333	198.812333	319.197667	358.099333
Pkor01G01489	*PkGLP1-3-2*	*37.017*	44.2396667	33.2636667	24.444	25.2563333	34.9753333	30.9133333	37.222	38.618
Pkor01G01496	*PkGLP3-2-7*	*0*	0.01433333	0	0.015	0.029	0.01433333	0	0.014	0
Pkor02G00787	*PkGLP5-15-1*	*19.858*	19.5166667	11.0286667	9.049	6.65133333	27.4663333	8.62933333	15.7803333	15.05
Pkor02G00794	*PkGLP5-15-2*	*49.8976667*	56.082	42.2466667	20.455	22.17	54.7596667	17.0806667	31.1976667	34.3676667
Pkor03G00261	*PkGLP1-2-1*	*0*	0.02	0	0	0	0	0	0	0
Pkor03G02030	*PkGLP3-7-5*	*0.08966667*	0.36466667	0.33266667	0.24266667	0.20266667	0.931	0.384	0.32666667	0.287
Pkor04G01078	*PkGLP1-2-3*	*0.01266667*	0.038	0.01266667	0.08733333	0.03833333	0.063	0	0.01233333	0
Pkor04G01177	*PkGLP5-5-3*	*0.01666667*	0.201	0.034	0	0.05066667	0.234	0.082	0.066	0.06633333
Pkor04G01178	*PkGLP1-2-4*	*0*	0.242	0.03266667	0.04833333	0.03266667	0.24166667	0.06333333	0.06466667	0.06366667
Pkor04G01179	*PkGLP1-2-5*	*0.07466667*	1.02	0.177	0.45333333	0.13233333	1.69	0.42766667	0.54266667	0.403
Pkor04G01180	*PkGLP1-2-6*	*0.06833333*	0.66733333	0.122	0.26666667	0.161	1.277	0.28833333	0.35933333	0.35566667
Pkor04G01181	*PkGLP1-2-7*	*0.53133333*	2.92033333	1.22166667	1.06833333	0.61266667	5.23066667	1.153	1.373	2.38066667
Pkor04G01182	*PkGLP1-2-8*	*0.03766667*	0.48166667	0.16233333	0.13633333	0.161	1.254	0.25433333	0.18533333	0.25633333
Pkor04G01183	*PkGLP1-2-9*	*0*	0	0	0	0	0.02666667	0	0	0
Pkor04G01186	*PkGLP1-2-11*	*0.01366667*	0	0.01433333	0.014	0.04133333	0.01366667	0.013	0.01366667	0.027
Pkor04G01187	*PkGLP5-14-1*	*0.038*	0	0.01233333	0.012	0	0.03633333	0.04766667	0.012	0.06033333
Pkor04G01188	*PkGLP5-14-2*	*0.04266667*	0.02666667	0	0.01333333	0	0.10666667	0	0.01366667	0
Pkor04G01189	*PkGLP5-14-3*	*0.02433333*	0.07333333	0.101	0.04966667	0.074	0.049	0.10833333	0.08633333	0.097
Pkor04G01190	*PkGLP1-2-12*	*0.013*	0.02566667	0.01366667	0.026	0.026	0.013	0.02566667	0.02566667	0.039
Pkor04G01191	*PkGLP1-2-13*	*0.06333333*	0.02466667	0.05166667	0.025	0	0.02466667	0.04833333	0.02466667	0.074
Pkor04G01192	*PkGLP1-2-14*	*0.06233333*	0.04966667	0.06266667	0.025	0.01233333	0.06166667	0.03666667	0.02433333	0.036

Table S3 Expression analysis of *PkGLP* gene during the maturation of lower body embryos under different concentrations of gellan gum(continued)

Gene ID	Gene ID	ck	4g-3h	12g-3h	4g-6h	12g-6h	4g-12h	12g-12h	4g-24h	12g-24h
Pkor04G01194	*PkGLP1-2-15*	*0*	0	0.03133333	0.031	0	0.015	0.01466667	0.03	0
Pkor04G01195	*PkGLP1-2-16*	*0*	0	0	0	0	0	0.012	0	0
Pkor04G01198	*PkGLP1-2-19*	*0.013*	0	0.01333333	0	0.01333333	0.026	0.01266667	0.013	0.02633333
Pkor04G01199	*PkGLP1-2-20*	*0*	0.063	0.016	0	0.01566667	0.094	0.015	0.03166667	0
Pkor04G01200	*PkGLP1-2-21*	*0.03933333*	0.78666667	0.34566667	0.27533333	0.14466667	1.84533333	0.47633333	0.564	0.74866667
Pkor04G01203	*PkGLP5-14-5*	*0*	0.02566667	0.01333333	0.03833333	0.013	0.01266667	0.02533333	0	0.013
Pkor04G01341	*PkGLP3-7-6*	*0.01366667*	0.05433333	0.12566667	0	0.04133333	0	0.02633333	0	0
Pkor04G01342	*PkGLP1-2-22*	*0*	0	0	0	0	0	0	0	0
Pkor04G01343	*PkGLP3-7-7*	*0*	0.026	0.01366667	0	0	0	0	0	0
Pkor04G02669	*PkGLP1-2-23*	*0*	0.04166667	0.014	0	0.014	0	0	0	0
Pkor04G02674	*PkGLP1-2-28*	*0*	0.02766667	0	0	0.014	0	0	0	0
Pkor04G02675	*PkGLP1-2-29*	*0*	0.014	0.014	0	0	0	0	0	0
Pkor04G02677	*PkGLP1-2-31*	*0*	0	0	0	0.01466667	0	0	0	0
Pkor04G02679	*PkGLP1-2-33*	*0*	0.014	0	0	0	0	0	0	0
Pkor04G02686	*PkGLP1-2-39*	*0*	0.01533333	0	0	0	0	0	0	0
Pkor04G02689	*PkGLP1-2-41*	*0*	0.014	0	0	0	0	0	0	0
Pkor04G02690	*PkGLP1-2-42*	*0*	0	0	0	0	0	0	0.01566667	0
Pkor04G02696	*PkGLP1-2-46*	*0*	0.05533333	0.014	0.014	0	0.01366667	0	0.014	0
Pkor06G01229	*PkGLP1-4*	*1.02233333*	4.314	3.09866667	2.726	1.63266667	3.81866667	3.29366667	2.81166667	2.625
Pkor06G01230	*PkGLP1-2-49*	*1.16*	1.13	1.111	1.27266667	1.036	1.366	0.94	1.049	1.81066667
Pkor06G01233	*PkGLP1-2-50*	*0.04533333*	0.44966667	0.147	0.188	0.08733333	0.33333333	0.129	0.05833333	0.02833333
Pkor06G01558	*PkGLP5-1-1*	*0*	0.01433333	0.01466667	0	0.01466667	0.02933333	0	0.029	0.014
Pkor06G01559	*PkGLP5-1-2*	*2.89533333*	6.066	4.32566667	2.59066667	2.735	5.34466667	1.76933333	4.08066667	4.21866667
Pkor08G02058	*PkGLP1-3-3*	*2.24266667*	2.47	4.12633333	3.30666667	3.57166667	3.049	3.464	3.36233333	2.84633333
Pkor08G02059	*PkGLP1-3-4*	*3.01366667*	3.266	4.13933333	4.64233333	4.26233333	3.748	3.78366667	3.93733333	3.45333333
Pkor08G02061	*PkGLP1-3-5*	*1.458*	1.46333333	1.987	1.41333333	1.719	1.52133333	1.44866667	1.363	1.32766667

Table S3 Expression analysis of *PkGLP* gene during the maturation of lower body embryos under different concentrations of gellan gum(continued)

Gene ID	Gene ID	ck	4g-3h	12g-3h	4g-6h	12g-6h	4g-12h	12g-12h	4g-24h	12g-24h
Pkor08G02062	*PkGLP1-3-6*	*0.884*	0.87	1.06233333	1.066	1.11933333	0.85633333	1.12366667	1.12233333	0.821
Pkor08G02063	*PkGLP1-3-7*	*0.04133333*	0.11033333	0.07066667	0.08433333	0.06966667	0.06866667	0.09533333	0.05566667	0.06833333
Pkor08G02064	*PkGLP1-3-8*	*0.026*	0.03866667	0.01366667	0	0.01333333	0.026	0.02533333	0.02533333	0.06433333
Pkor12G00734	*PkGLP3-8*	*64.4033333*	65.752	44.5443333	35.613	39.395	70.52	35.5703333	58.9343333	57.7466667

Table S4 Primer sequences

Primer	Sequence (5'to3')
PkGLP3-7-4-F	CGCTCTCCAAACCCTGCA
PkGLP3-7-4-R	CAGGGTCTGCTGGTTGGG
PkGLP1-3-2-F	TCCTCCACGAAGCGCAAG
PkGLP1-3-2-R	TTCGTTTCCCGGGCTGTC
PkGLP5-15-1-F	GTGAGCCGGGCAAGAACT
PkGLP5-15-1-R	TCCCCCTTCTACTGCCCC
PkGLP5-5-3-F	TGTGTTCTGATGCGCGGT
PkGLP5-5-3-R	CCTAGAGAAGCGCCACCG
PkGLP1-2-6-F	TCGGAGGTGTCGATGGGT
PkGLP1-2-6-R	TCCGTTCTCGCATCGACG
PkGLP1-2-21-F	AGCACCTCCGAGGATGGA
PkGLP1-2-21-R	CGCATGTGTTGGGAGGACT
PkGLP3-8-F	CCACCAGCTGCAGGAACA
PkGLP3-8-R	GCTGCAGCAGGTCCAGAA
GAPDH-F	CGGCGCAGAGTATGTGGT
GAPDH-R	TAGGCGCATCCTTGCTGG

Table S5 The cloning primer sequences of *PkGLP1-2-6* and *PkGLP1-2-21*

Primer	Sequence (5'to3')
PkGLP1-2-6-F	TCACATACATCCGCGGGC
PkGLP1-2-6-R	GCACAAGGCCTTTGGGGA
PkGLP1-2-21-F	CGCGCAGGTGATCCAGAT
PkGLP1-2-21-R	GCTTGCAGGGGAATCCGT
